# Distributed biotin–streptavidin transcription roadblocks for mapping cotranscriptional RNA folding

**DOI:** 10.1093/nar/gkx233

**Published:** 2017-04-08

**Authors:** Eric J. Strobel, Kyle E. Watters, Yuri Nedialkov, Irina Artsimovitch, Julius B. Lucks

**Affiliations:** 1 Department of Chemical and Biological Engineering, Northwestern University, Evanston, IL 60201, USA; 2 Department of Molecular and Cell Biology, University of California, Berkeley, CA 94720, USA; 3 Department of Microbiology, The Ohio State University, Columbus, OH 43210, USA; 4 The Center for RNA Biology, The Ohio State University, Columbus, OH 43210, USA

## Abstract

RNA folding during transcription directs an order of folding that can determine RNA structure and function. However, the experimental study of cotranscriptional RNA folding has been limited by the lack of easily approachable methods that can interrogate nascent RNA structure at nucleotide resolution. To address this, we previously developed cotranscriptional selective 2΄-hydroxyl acylation analyzed by primer extension sequencing (SHAPE-Seq) to simultaneously probe all intermediate RNA transcripts during transcription by stalling elongation complexes at catalytically dead EcoRI_E111Q_ roadblocks. While effective, the distribution of elongation complexes using EcoRI_E111Q_ requires laborious PCR using many different oligonucleotides for each sequence analyzed. Here, we improve the broad applicability of cotranscriptional SHAPE-Seq by developing a sequence-independent biotin–streptavidin (SAv) roadblocking strategy that simplifies the preparation of roadblocking DNA templates. We first determine the properties of biotin–SAv roadblocks. We then show that randomly distributed biotin–SAv roadblocks can be used in cotranscriptional SHAPE-Seq experiments to identify the same RNA structural transitions related to a riboswitch decision-making process that we previously identified using EcoRI_E111Q_. Lastly, we find that EcoRI_E111Q_ maps nascent RNA structure to specific transcript lengths more precisely than biotin–SAv and propose guidelines to leverage the complementary strengths of each transcription roadblock in cotranscriptional SHAPE-Seq.

## INTRODUCTION

The capacity for RNA to fold into sophisticated structures is integral to its roles in diverse cellular processes including gene expression, macromolecular assembly, and RNA splicing (1,2). Because RNA folding can occur on a shorter timescale than nucleotide addition by RNA polymerase (RNAP) (3–5), a nascent RNA can transition through multiple intermediate structural states as it is synthesized (6). Pioneering experimental studies of RNA cotranscriptional folding used biochemical methods to characterize these RNA structural intermediates (6–8) and more recently, single-molecule force spectroscopy has been used to directly observe RNA folding during transcription by measuring changes in RNA extension in real time (9). However, the lack of a robust method to directly interrogate RNA structure at nucleotide resolution during transcription has so far limited our ability to fully investigate the fundamental principles of RNA cotranscriptional folding and its impact on generating functional RNA structural states that govern biological processes.

We recently addressed this technological gap by developing cotranscriptional SHAPE-Seq to measure nascent RNA structures at nucleotide resolution (10). SHAPE-Seq combines chemical RNA structure probing with high-throughput sequencing to simultaneously characterize the structure of many RNAs in a mixture (11–13). Chemical modification of a target RNA is accomplished using any of the suite of SHAPE probes available that react with the RNA 2΄-hydroxyl at ‘flexible’ regions of the molecule, such as unpaired nucleotides in single stranded regions and loops (14). After reverse transcription (RT), modified nucleotides can be detected as truncated RT products using high-throughput sequencing. The resulting sequencing reads are then used to generate a ‘reactivity’ value for each nucleotide in each RNA (15). SHAPE-Seq reactivity represents the relative flexibility of each nucleotide of an RNA: highly reactive nucleotides tend to be single-stranded, whereas nucleotides with low reactivity tend to be involved in base-pairing or other intra- or intermolecular interactions (11,16).

Cotranscriptional SHAPE-Seq combines the ability of SHAPE-Seq to characterize complex mixtures of RNAs with *in vitro* transcription in order to simultaneously interrogate the structure of all intermediate lengths of a target RNA. Each intermediate length is probed in the context of stalled transcription elongation complexes (TECs) (10) which are generated by constructing a DNA template library containing a promoter, a variable length of the target RNA template, and an EcoRI recognition site. Within 30s of the start of *in vitro* transcription, TECs are blocked by a catalytically dead EcoRI_E111Q_ mutant (Gln111) (17,18) bound to the EcoRI recognition sites (10) and treated with either the fast acting SHAPE reagent benzoyl cyanide (BzCN, t_1/2_ of 250 ms) (19) or dimethyl sulfoxide (DMSO) as a control. RNAs are then quickly extracted and processed for paired-end sequencing to identify the transcript length and SHAPE-modification position as described previously (10,11). RNA structural states that persist on the order of seconds are interrogated to provide ‘snapshots’ of kinetically trapped intermediates that reveal key transitions within RNA folding pathways (10).

The cotranscriptional SHAPE-Seq experiment requires stalled TECs to be generated for all intermediate lengths within a target RNA sequence at once. Gln111 was initially selected as a roadblock because its ability to halt *Escherichia coli* RNAP is both robust and well characterized (18). However, the use of Gln111 comes with a number of drawbacks. Constructing the Gln111 DNA template library requires a unique primer set that encodes every stop for each RNA target and therefore contributes substantially to experimental costs. This is exacerbated during mutational analysis as many additional primers are required in order to preserve the mutation in the DNA template library. Furthermore, intermediate lengths that are poorly amplified create the potential for gaps in the experimental data, which is particularly problematic for highly repetitive sequences. Lastly, RNA sequences that contain an internal EcoRI recognition site ‘GAATTC’ must be mutated to access structural information downstream or an alternative transcription roadblock (20,21) must be used. Thus, the development of a sequence-independent roadblocking strategy is highly desirable in order to reduce both experimental costs and time, thereby facilitating a broad application of cotranscriptional SHAPE-Seq to the study of how RNA folding directs RNA function.

**Figure 1.**
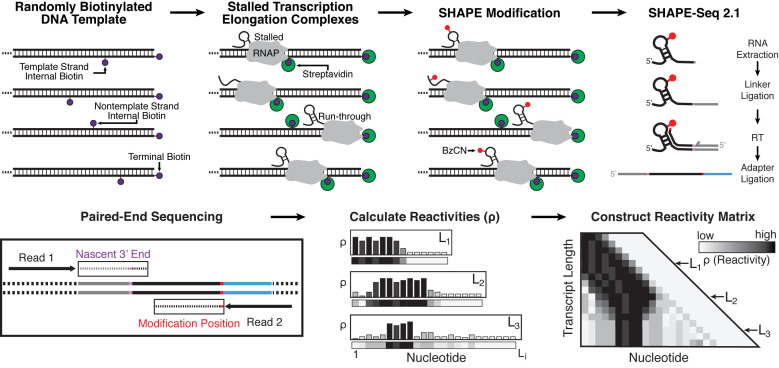
Overview of biotin–streptavidin (SAv) roadblocking in cotranscriptional SHAPE-Seq. DNA templates containing random internal biotin modifications and a 5΄ terminal biotin modification on the template strand are prepared by PCR amplification. Following open complex formation and incubation with SAv, single-round transcription is initiated to generate stalled transcription elongation complexes. After 30s, nascent RNAs are treated with either BzCN (+) or DMSO (−), extracted and processed for paired end sequencing using the SHAPE-Seq v2.1 protocol (11). Transcript length and SHAPE modification position are identified by paired-end sequencing and used to calculate SHAPE reactivity profiles for each length of the nascent RNA. Individual reactivity profiles are stacked to generate a reactivity matrix. The ‘SHAPE-Seq 2.1’, ‘Paired-End Sequencing’, ‘Calculate Reactivities’ and ‘Construct Reactivity Matrix’ panels are adapted from (10).

Here we develop a sequence-independent method for halting TECs at all positions across a DNA template using streptavidin (SAv) as a transcription roadblock and combine this method with SHAPE-Seq to characterize cotranscriptional RNA folding pathways at nucleotide resolution (Figure 1). We start by characterizing the robustness of biotin–SAv transcription roadblocks in the context of *in vitro* transcription and perform a rigorous analysis of how collision with a roadblock influences RNAP position. We then implement biotin–SAv roadblocking in the cotranscriptional SHAPE-Seq framework using randomly biotinylated DNA templates to capture TECs across all transcript lengths in a general workflow that can be applied to any RNA sequence. A comparison of the SAv and Gln111 roadblocking strategies using the *Bacillus cereus crcB* fluoride riboswitch (22) as a model system allowed us to determine how technical distinctions between biotin–SAv and Gln111 roadblocking can influence cotranscriptional SHAPE-Seq data. Finally, we propose experimental strategies that leverage the complementary strengths of each approach. The robust and sequence-independent nature of biotin–SAv roadblocking is a powerful addition to the cotranscriptional SHAPE-Seq method that uses reagents that are all commercially available, reduces experimental costs, and simplifies materials preparation. Together, these improvements increase the accessibility of cotranscriptional SHAPE-Seq to a broader user base to study cotranscriptional RNA folding.

## MATERIALS AND METHODS

### DNA template preparation

#### Preparation of J23119 DNA templates

DNA templates for *in vitro* radiolabeled transcription experiments were prepared by PCR amplification. 500 μl reactions included 411.25 μl H_2_O, 50 μl 10× ThermoPol Buffer (New England Biolabs), 6.25 μl 10 mM dNTPs, 12.5 μl 10 μM forward primer (Supplementary Table S1), 12.5 μl 10 μM reverse primer (Supplementary Table S1), 2.5 μl template plasmid DNA and 5 μl of Vent Exo-DNA polymerase (New England Biolabs). 100 μl aliquots were amplified with a thermal cycling program consisting of 30 cycles using an annealing temperature of 55°C. After thermal cycling, reactions were pooled into two 250 μl aliquots, mixed with 50 μl 3 M sodium acetate (NaOAc) pH 5.5 and 1 ml 100% ethanol (EtOH) each, and stored at −80°C for 30 min. After centrifugation, precipitated pellets were washed with 1.5 ml cold 70% EtOH, and dried using a SpeedVac. Dried pellets were pooled by dissolving in 30 μl H_2_O, fractionated by gel electrophoresis with a 1% agarose gel, and extracted using the QIAquick Gel Extraction Kit (Qiagen). Purified template was quantified using a Qubit Fluorometer (Life Technologies). Amplification of SRP DNA templates (Supplementary Table S2) with a biotin modification at positions 33 and 42 relative to the transcription start site was directed with oligonucleotides A and B or C (Supplementary Table S1), respectively.

To generate a DNA template containing a single nontemplate strand biotin modification, 10 μM oligonucleotide D and 10 μM oligonucleotide E (Supplementary Table S1) in 50 μl of 1× ThermoPol buffer (New England Biolabs) was incubated at 95°C for 5 min and annealed by incubating at 37°C for 20 min. After chilling annealed oligonucleotides at 4°C for 1 min, 10 U of ExoI was added and the sample was incubated at 37°C for 30 min to remove excess oligonucleotides. The resulting DNA templates were first purified by using the QIAquick Purification Kit (QIAgen) and then run on a 1% agarose gel and gel extracted using the QIAquick Gel Extraction Kit (Qiagen). Purified DNA template concentration was measured using a Qubit Fluorometer (Life Technologies).

#### Preparation of λ P_R_ templates containing precisely positioned roadblocks

Linear DNA templates were generated by PCR amplification of pIA226 plasmid with Taq1 DNA polymerase (New England Biolabs). A common upstream primer (Supplementary Table S1, oligonucleotide H) was used with downstream primers (Integrated DNA Technologies) containing a desired modification: an EcoRI recognition site (Supplementary Table S1, oligonucleotide I) or an internal biotin-dT (Supplementary Table S1, oligonucleotide J). The EcoRI site and internal biotin-dT were positioned to roadblock RNAP at the same nucleotide. For ExoIII footprinting, the template (bottom) strand primers were end-labeled with [γ^32^P]-ATP using PNK (New England Biolabs) and purified using G-50 spin columns (GE Healthcare).

#### Preparation of Biotinylated DNA Templates

Randomly biotinylated DNA templates were prepared by PCR amplification and gel extraction as described above except that instead of supplying a dNTP mixture, each dNTP was added individually to a total of 100 nmol combined dNTP and biotin-11-dNTP. Assuming equal probability of incorporating a biotinylated or non-biotinylated dNTP, for 1× biotin incorporation, the nmol quantity of each biotin-11-dNTP included in the reaction was determined using the formula:

\begin{equation*}{{\rm dNTP}_{\rm bio}} = \frac{{{\rm dNTP}_{\rm comb}}}{{4{N_{\rm count}}}}\end{equation*}

where dNTP_bio_ is the nmol quantity of biotin-11-dNTP for base *N* included in the reaction, *N*_count_ is number of occurrences of base *N* in the template and nontemplate strands of the DNA sequence that encodes the target RNA (not including reverse primer sequence), and dNTP_comb_ is the combined nmol quantity biotinylated and non-biotinylated dNTP for base *N* included in the reaction fixed by the PCR condition. For increased biotin modification, dNTP_bio_ was then multiplied by the desired number of biotin modifications per template. The quantity of each non-biotinylated dNTP included in the reaction was determined by subtracting dNTP_bio_ from dNTP_comb_. Biotin-11-dATP and biotin-11-dGTP were purchased from PerkinElmer. Biotin-11-dCTP and biotin-11-dUTP were purchased from Biotium. Amplification of randomly biotinylated *B. cereus crcB* fluoride riboswitch DNA templates (Supplementary Table S2) was directed by oligonucleotides F and G (Supplementary Table S1).

### *In vitro* transcription (radiolabeled)

For each sample, 0.125 pmol of biotinylated DNA template was pre-incubated with 12.5 pmol SAv monomer (Promega) at room temperature for 30 min. 25 μl reaction mixtures containing 5 nM DNA template/SAv complexes and 0.5 U RNAP Holo (New England Biolabs) were incubated in transcription buffer (20 mM tris(hydroxymethyl)aminomethane hydrochloride (Tris–HCl) pH 8.0, 0.1 mM ethylenediaminetetraacetic acid (EDTA), 1 mM dithiothreitol (DTT) and 50 mM potassium chloride (KCl), 0.2 mg/ml bovine serum albumin (BSA), and 200 μM ATP, GTP, CTP and 50 μM UTP containing 0.5 μCi/μl [α-^32^P]-UTP for 10 min at 37°C to form open complexes. Single-round transcription was initiated by the addition of magnesium chloride (MgCl_2_) to 5 mM and rifampicin to 10 μg/ml. Transcription was stopped by adding 125 μl of stop solution (0.6 M Tris pH 8.0, 12 mM EDTA, 0.16 mg/ml tRNA).

For pellet/supernatant separation experiments, 10 μl of streptavidin magnesphere paramagnetic particles (Promega) per sample were first prepared by washing three times with SAv binding buffer (0.5 M sodium chloride (NaCl) and 20 mM Tris–HCl pH 7.5) before incubating with 0.125 pmol template DNA per sample in SAv binding buffer for 30 min. Bound templates were pulled-down and washed three times with 1× transcription buffer. *In vitro* transcription reactions were mixed as described above. After 30s, reactions were placed on a magnetic stand and allowed to separate for 30s before the supernatant was removed and added to 125 μl of stop solution and the pellet was resuspended in 125 μl of stop solution and 25 μl 1× transcription buffer.

Following *in vitro* transcription, RNA was purified by the addition of 150 μl of phenol/chloroform/isoamyl alcohol (25:24:1), vortexing, centrifugation and collection of the aqueous phase. RNA was precipitated by adding 450 μl of 100% EtOH and storage at −20°C. Following precipitation, RNA was resuspended in transcription loading dye (1× transcription buffer, 80% formamide, 0.05% bromophenol blue and xylene cyanol). RNAs were fractionated by electrophoresis using 12% denaturing polyacrylamide gels containing 7.5 M urea (National Diagnostics, UreaGel). Reactive bases were detected using an Amersham Biosciences Typhoon 9400 Variable Mode Imager. Quantification of bands was performed using ImageQuant. For all experiments, individual bands were normalized for incorporation of [α-^32^P]-UTP by dividing band intensity by the number of U's in the transcript. biotin–SAv roadblocking efficiency was calculated by dividing the sum of roadblocked RNAs by the sum of all roadblocked and run-off products. Aborted/paused products are not included in this calculation.

### GreB cleavage assay

Linear DNA templates (40 nM), holo-RNAP (80 nM), ApU (100 μM) and starting NTPs (1 μM UTP, 5 μM GTP and ATP, 8 μCi [α^32^P]-UTP, 3000 Ci/mmol) were mixed in TB-50 (20 mM Tris–HCl, 50 mM KCl, 2 mM MgCl_2_, 5% glycerol, 0.1 mM EDTA, 1 mM β-mercaptoethanol, pH 7.9). Reactions were incubated for 10 min at 37°C to form a halted A26 TEC. 500 nM Gln111 and 1.5 μM SAv were added to templates containing the EcoRI recognition sequence and biotinylated TMP respectively, and incubated for 8 min at 37°C. Transcription was restarted by the addition of all NTPs at 200 μM and 100 nM GreB, where indicated, and incubated at 37°C. At times indicated in the figure, the reactions were quenched with an equal volume of Stop buffer [8 M urea, 20 mM EDTA, 1× Tris/borate/EDTA (TBE), 0.5% Brilliant Blue R and 0.5% Xylene Cyanol FF] and analyzed on 15% polyacrylamide–7 M urea gels.

### Exonuclease footprinting

Linear radiolabeled DNA templates (140 nM), holo-RNAP (160 nM), ApU (100 μM) and 5 μM each UTP, GTP and ATP were incubated in TB-50 for 10 min at 37°C to form a halted A26 TEC. 500 nM Gln111 or 1.5 μM SAv were added to DNA-RI and DNA-B respectively and incubated for 8 min at 37°C. Transcription was restarted by the addition of all NTPs at 200 μM, samples were aliquoted at 5 μl/tube. ExoIII (New England Biolabs) was diluted in TB-50 and added to the stalled complexes at a final concentration of 4 U/μl for 3 min at 21°C. Reactions were quenched with the Stop buffer and analyzed on a 6% polyacrylamide–7 M urea gel.

### Electrophoretic mobility shift assay

Randomly biotinylated DNA templates were prepared as described above except that an unmodified reverse primer (Supplementary Table S1, oligonucleotide K) was used. 1 pmol of DNA template was incubated in the absence or presence of 10 pmol SAv for 15 min at room temperature. Six times Purple Gel Loading Dye (no SDS) (New England Biolabs) was added and samples were fractionated in using a 1.5% Agarose gel containing 1× GelRed (Biotium). Bands were detected by UV transillumination using a ChemiDoc (Bio-Rad).

### *In vitro* transcription (cotranscriptional SHAPE-Seq)

Reaction mixtures containing 100 nM randomly biotinylated DNA template and 4 U of *E. coli* RNAP holoenzyme (New England Biolabs) were incubated in transcription buffer, 0.2 mg/ml BSA, and 500 μM NTPs for 7.5 min at 37°C to form open complexes. When present, NaF was included to a final concentration of 10 mM. Following open complex formation, SAv monomer (Promega) was added to 40 μM and incubated for another 7.5 min. Single-round transcription reactions were initiated by addition of MgCl_2_ to 5 mM and rifampicin to 10 μg/ml. After 30s, RNAs were SHAPE modified by splitting the reaction and mixing half with 2.78 μl of 400 mM BzCN dissolved in anhydrous DMSO (+) sample) or anhydrous DMSO only (Sigma Aldrich; (−) sample) for ∼2s before addition of 75 μl of TRIzol solution (Life Technologies) and extraction. Extracted RNAs were dissolved in 20 μl 1× DNase I buffer (New England Biolabs) containing 1 U DNase I (New England Biolabs) and incubated at 37°C for 30 min. After DNA digestion, 30 μl of H_2_O and 150 μl of TRIzol were added and the RNA was extracted and dissolved in 10 μl 10% DMSO.

### Sequencing library processing

An RNA linker was adenylated using the 5΄ DNA adenylation kit (New England Biolabs), purified by TRIzol extraction, and quantified using a Qubit Fluorometer as described previously (10). Extracted RNAs were ligated to an RNA linker using T4 RNA Ligase 2 truncated KQ (New England Biolabs) by incubation at room temperature as described previously (10). Reverse transcription of the linker ligation products was performed using Superscript III Reverse Transcriptase (Life Technologies) as described previously (10). Ligation of an Illumina A_b adapter fragment was performed using CircLigase I ssDNA ligase (Epicentre) as described previously (10). ssDNA libraries were used to generate fluorescently labeled dsDNA libraries as described previously (10). The resulting dsDNA libraries were analyzed by capillary electrophoresis using an ABI 3730xl and the resulting traces were used to evaluate library length distribution and the presence of adapter dimer prior to sequencing. Sequencing libraries were generated as described previously (11).

### Sequencing and analysis

Sequencing of 1× (replicate 1), 2× and 4× biotin fluoride riboswitch cotranscriptional SHAPE-Seq libraries was performed on the Illumina HiSeq2500 in Rapid Run mode using 2 × 36 bp paired end reads and 20% phiX. 1× biotin fluoride riboswitch replicates 2–4 were performed in a completely different laboratory setting using completely different reagents and sequenced on the Illumina NextSeq500 using 2 × 37 bp paired end reads and 30% phiX. All cotranscriptional SHAPE-Seq computational tools used in this study can be found on GitHub at: https://github.com/LucksLab/Cotrans_SHAPE-Seq_Tools/releases/ and https://github.com/LucksLab/spats/releases/. Target FASTA files were prepared using the Cotrans_targets.py script as described previously (10). Reads were mapped and processed for Spats v1.0.1 as described previously (11). In general, between 4 and 6 M reads (samples run on HiSeq2500) or between 9 and 16 M reads (samples run on NextSeq500) were mapped and used to calculate the reported cotranscriptional reactivity matrices.

## RESULTS

### The efficiency of streptavidin transcription roadblocking is DNA strand dependent

Biotin–SAv roadblocking (23,24) has previously been shown to prevent *E. coli* RNAP from ‘running off’ the DNA template during *in vitro* transcription (9). Typically, biotin–SAv roadblocks are introduced into a DNA template to prevent run-off transcription by including a 5΄-biotinylated reverse primer during template amplification (25). Recently, a terminal biotin–SAv roadblock was used to halt a TEC to facilitate SHAPE probing of a single RNA transcript in complex with RNAP (26). Cotranscriptional SHAPE-Seq, however, requires the distribution of stalled TECs across all DNA template positions. Thus, the use of biotin–SAv roadblocking in cotranscriptional SHAPE-Seq requires random biotinylation of the DNA template during PCR, which inherently biotinylates both the template and nontemplate strands of the DNA duplex. Because each DNA strand makes distinct interactions with RNAP, a biotin–SAv roadblock in the template strand may not have an equivalent effect on RNAP compared to the nontemplate strand.

**Figure 2.**
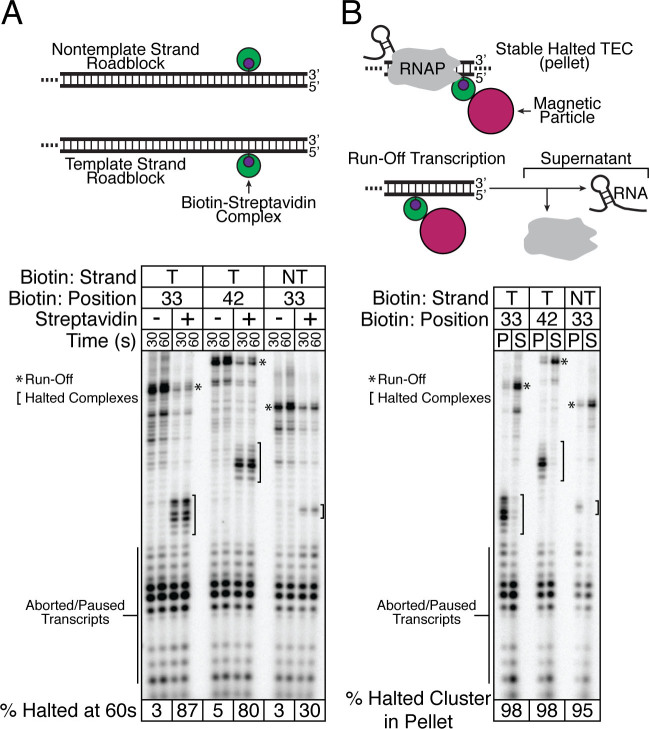
Characterization of biotin–SAv transcription roadblocks. (**A**) Template strand biotin–SAv complexes roadblock TECs with greater efficiency than nontemplate strand biotin–SAv complexes. Single-round *in vitro* transcription was performed using DNA templates encoding a fragment of the *E. coli* signal recognition particle (SRP) RNA with a template or nontemplate strand biotin modification in the absence and presence of SAv. The distribution of roadblocked TECs does not change between 30 and 60s. The percentage of complexes roadblocked by biotin–SAv at 60s is shown. A * indicates run-off transcripts while a bracket indicates roadblocked complexes. (**B**) TECs are not dissociated by collision with a biotin–SAv roadblock. The DNA templates described in (A) were immobilized on SAv-coated magnetic particles to facilitate fractionation of single-round *in vitro* transcription reactions. RNAs within roadblocked TECs are present in the pellet (P), whereas free RNAs are in the supernatant (S). The percentage of roadblocked TEC RNAs present in the pellet compared to the supernatant is shown. Virtually all RNAs associated with roadblocked TECs are partitioned into the pellet, indicating that TECs stalled at biotin–SAv roadblocks are stable.

To measure the efficiency of template vs. nontemplate strand biotin–SAv roadblocks, we performed *in vitro* transcription using DNA templates that were biotinylated at a single internal position downstream of the promoter in the template strand (positions +33 or +42) or in the nontemplate strand (position +33) (Figure 2A). Template strand biotin–SAv roadblocks stall TECs with 80–87% efficiency as a cluster of stops 7–13 nucleotides (nts) upstream of the biotinylation site. In contrast, nontemplate strand biotin–SAv roadblocks stall TECs as a more defined stop, but with only 30% efficiency (Figure 2A). The superior roadblocking efficiency of the template strand biotin–SAv roadblocks is consistent with the trajectory of each DNA strand through RNAP: the template strand must traverse the RNAP active site deep within the polymerase internal channel, while the nontemplate strand is less constrained (27). The clustered distribution of TECs stalled by a biotin–SAv roadblock is distinct from the defined stop 14 nt upstream of the EcoRI for TECs stalled by Gln111 (18), and is likely due to the flexible linker between biotin and the DNA template.

### Collision of RNAP with a streptavidin roadblock does not dissociate TECs

Because cotranscriptional SHAPE-Seq aims to probe RNAs in the context of a TEC, it is critical that collision of RNAP with a roadblock does not dissociate the TEC and release the RNA. To assess the stability of TECs stalled at an internal biotin–SAv roadblock, we immobilized each biotinylated template using SAv paramagnetic beads and separated RNAs in complex with a stable TEC from free RNAs by magnetic pull-down (Figure 2B). We observed that 95–98% of the RNAs in stalled TECs were still attached to beads after the pull-down, indicating that the vast majority of RNAs remain stably associated with RNAP in stalled TECs independent of the strand to which the biotin–SAv roadblock is tethered (Figure 2B).

### Collision with a streptavidin roadblock, but not Gln111, induces extensive RNAP backtracking

**Figure 3.**
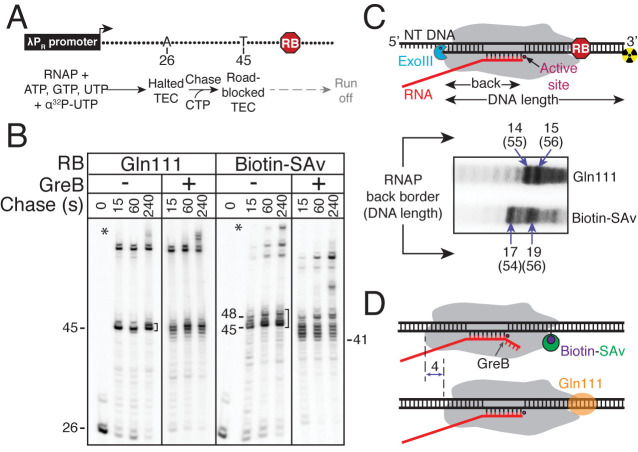
Determining the translocation register of RNAP stalled by Gln111 and biotin–SAv transcription roadblocks. (**A**) Halted radiolabeled A26 TECs were formed on linear λ P_R_ templates with attached roadblocks (RB) and (**B**) chased for indicated times in the absence or in the presence of *E. coli* GreB. On both templates, the majority of RNAPs stalled at the U45 position, with an additional cluster of positions observed for biotin–SAv roadblocks that are consistent with Figure 2. Roadblocked run-off RNAs are indicated as in Figure 2. Treatment with GreB reveals extensive backtracking of SAv-stalled RNAP observed as an increase in shorter length transcripts generated by GreB-mediated cleavage of backtracked complexes. (**C**) ExoIII footprinting of TECs stalled on RB templates with an end-labeled template DNA strand. The extent of the DNA protected from ExoIII allows the distance between the RNAP back border and the 3΄ end of the nascent RNA to be inferred. Note that the Gln111 template is 4 bp longer than the biotin–SAv template. This difference in DNA template length was accounted for when converting the observed template strand DNA length to the location of the RNAP back end border (Supplementary Note S1). (**D**) Summary of the RNA cleavage and ExoIII probing results indicating that Gln111 produces a robust roadblocked complex while biotin–SAv causes up to a 4 bp RNAP backtrack.

The observation that template strand biotin–SAv roadblocks stall RNAP at a range of positions suggests that the enzyme may reverse translocate (backtrack) (28) upon collision with a flexibly attached roadblock. By contrast, RNAP may remain stationary after running into a rigid Gln111 roadblock. Knowing the RNAP position on the nascent RNA is essential for interpreting the RNA modification patterns. To compare the structures of TECs stalled by the two roadblocks, we designed templates on which RNAP was stalled at the same position by a biotin in the template strand or Gln111 bound to GAATTC (Figure 3A). On these templates, RNAP that initiates transcription from a strong bacteriophage λ P_R_ promoter in the absence of CTP is halted after incorporating the A residue at position 26. In the presence of all NTPs, RNAP resumes elongation and is efficiently stalled by either Gln111 or biotin–SAv linked to the template DNA strand (Figure 3B). An EcoRI site and internal biotinylated dT were positioned to achieve RNAP stalling at the same position on the respective templates (after the addition of U45 on templates used in these experiments). As was observed in Figure 2, collision of RNAP with a biotin–SAv roadblock yields a cluster of roadblocked TECs across several transcript lengths.

To test if RNAP was backtracked after running into each roadblock, we used GreB, an *E. coli* elongation factor that stimulates the intrinsic endonucleolytic cleavage of the nascent RNA in backtracked TECs (29). We observed several GreB-induced cleavages in SAv-stalled complexes, consistent with RNAP backtracking by up to 4 nt upstream of the primary roadblock position at +45 (Figure 3B). Because some TECs transcribe to +48 by ‘pushing’ the biotin–SAv prior to reverse translocation, the maximum possible backtrack in this sequence context is 7 nt, however, such extensive backtracking is likely to be infrequent because only ∼25% of roadblocked TECs have transcribed to +47 or +48 and these complexes may backtrack to any position between +41 and +45. By contrast, Gln111-stalled TECs did not backtrack by more than 1 nt (Figure 3B). These results suggest that RNAP is shifted backwards in biotin–SAv-stalled complexes, as compared to those stalled by Gln111.

We next used exonuclease III (ExoIII) to map the back border of RNAP in stalled TECs (Figure 3C). ExoIII is a processive 3΄–5΄ exonuclease used extensively to determine the RNAP translocation register (30). The biotin–SAv-stalled RNAP protected 17–19 nts of the template DNA strand upstream of A45 from ExoIII cleavage, whereas Gln111-stalled RNAP protected only 14–15 nts upstream of A45 (Figure 3C). We conclude that when RNAP runs into a biotin–SAv roadblock, an additional 3–4 nts of the nascent RNA becomes protected inside the backtracked enzyme, as compared to the enzyme stalled by Gln111 (Figure 3D).

### Design of randomly biotinylated DNA templates for cotranscriptional SHAPE-Seq

**Figure 4.**
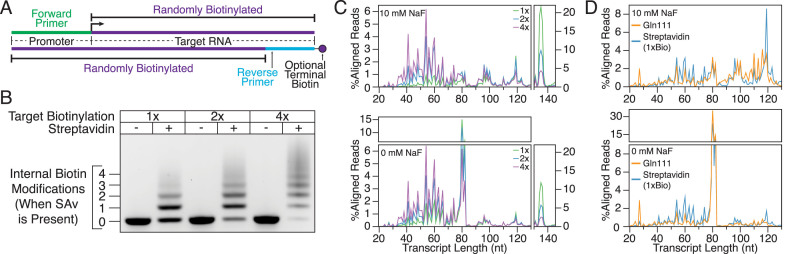
Distribution of aligned reads across transcript lengths. (**A**) Schematic of randomly biotinylated DNA templates used in cotranscriptional SHAPE-Seq experiments. (**B**) Gel shift assay showing the distribution in the number of internal biotins that are introduced during PCR with biotinylated dNTPs used at concentrations calculated to introduce an average of one, two or four biotins per DNA template. DNA templates used in this experiment did not contain a terminal biotin modification. (**C**) Distribution of read alignments over transcript lengths from cotranscriptional SHAPE-Seq with biotin–SAv roadblocking. Cotranscriptional SHAPE-Seq was performed on the *Bacillus cereus crcB* fluoride riboswitch with 10 mM or 0 mM fluoride using DNA templates with a targeted biotinylation rate of one, two, or four modifications per template. %Aligned Reads is calculated by dividing the unmodified RNA reads aligned at each transcript length by total unmodified reads aligned. ‘Full length’ terminally roadblocked TECs are broken from internal stops to provide a clear view of the accumulation of both classes of stops. Fluoride riboswitch function is evident in the distribution of RNAs transcribed without fluoride, which accumulate as terminated products from positions 80–82, compared to those transcribed with fluoride, which are distributed at positions beyond the terminator or accumulate at the terminal roadblock. (**D**) Comparison of read alignment distribution for biotin–SAv and Gln111 cotranscriptional SHAPE-Seq of the *Bacillus cereus crcB* fluoride riboswitch. Reads aligning to terminally roadblocked transcripts were not included when calculating the percentage of reads aligned to each transcript length since Gln111 roadblock run-through transcripts do not align due to the presence of an EcoRI recognition site in the DNA template. Gln111 cotranscriptional SHAPE-Seq data was downloaded from the Small Read Archive (http://www.ncbi.nlm.nih.gov/sra) (Supplementary Table S5).

Having established that biotin–SAv roadblocking can be used to halt RNAP in stable TECs, we next sought to validate its use in the cotranscriptional SHAPE-Seq experimental framework. Randomly biotinylated DNA templates were prepared by enzymatic incorporation of biotin-11-dNTPs during PCR amplification. Vent Exo- was selected for template amplification as it is particularly tolerant of biotin-11-dNTPs (31). To prevent biotinylation of the promoter nontemplate strand, which could interfere with promoter open complex formation, the forward PCR primer comprised positions –45 to –1 relative to the transcription start site (Figure 4A). Optionally, the reverse primer can include a 5΄ biotin modification as a terminal roadblock to prevent template run-off during the cotranscriptional SHAPE-Seq experiment (Figure 4A). We prepared randomly biotinylated DNA templates for the *B. cereus crcB* fluoride riboswitch (22) with targeted biotinylation levels of one, two, or four modifications per DNA template (Figure 4B) and performed cotranscriptional SHAPE-Seq in the presence and absence of fluoride. This choice of model system allowed us to compare the general characteristics of the biotin–SAv roadblocking strategy with our previous Gln111 approach (10), and to perform a detailed comparison of the reactivity profiles obtained from each method.

### Validation: Analysis of streptavidin and Gln111 transcript length alignments

To obtain a complete reactivity matrix for a target RNA, it is necessary to interrogate the structure of all intermediate length transcripts. Thus, it is critical that all RNA intermediates are well represented in cotranscriptional SHAPE-Seq libraries. To assess coverage of the intermediate transcript lengths when biotin–SAv roadblocks are randomly incorporated, we examined the *B. cereus crcB* fluoride riboswitch (22,32) with cotranscriptional SHAPE-Seq using varying degrees of biotinylation (1, 2 or 4 biotins/template) as described above. We then compared the distribution of unmodified transcript lengths to the number of biotins expected to be present in the DNA template (Figure 4C). Increased DNA template biotinylation proportionally shifts the distribution toward shorter lengths as it becomes increasingly likely for RNAP to encounter a biotin–SAv roadblock (Figure 4C). As an alternative to increased template biotinylation, enrichment for stalled complexes could also be achieved by omitting the terminal roadblock and using immobilized DNA templates to remove run-off transcripts before the transcription reaction is stopped (Figure 2B).

In all samples, the transcript lengths are distributed unevenly in a distinct pattern of peaks and troughs, representing high and low abundance, respectively. Interestingly, comparison of the alignment distributions produced by biotin–SAv and Gln111 roadblocking reveal remarkable consistency between both methods (Figure 4D). One plausible explanation for the similarity of biotin–SAv and Gln111 transcript distributions is that the linker ligation step used to facilitate reverse transcription in the SHAPE-Seq v2.1 strategy (11) influences the representation of transcript lengths in cotranscriptional SHAPE-Seq libraries. There is well-documented structure- and sequence-dependent bias (33,34) in RNA–RNA ligations. While such bias does not influence cotranscriptional SHAPE-Seq reactivity calculation, as reactivity profiles are calculated internally for each length, reduction of ligation bias is a target of future development as it would reduce the sequencing depth necessary to adequately cover all intermediate transcript lengths by flattening the transcript length distributions. Because the transcript length distribution of cotranscriptional SHAPE-Seq libraries produced using biotin–SAv roadblocking approximates that of libraries produced using Gln111, we conclude that biotin–SAv roadblocking provides a sufficient distribution of TECs for reliable cotranscriptional SHAPE-Seq measurements.

### Validation: analysis of streptavidin and Gln111 cotranscriptional SHAPE-Seq reactivity measurements

**Figure 5.**
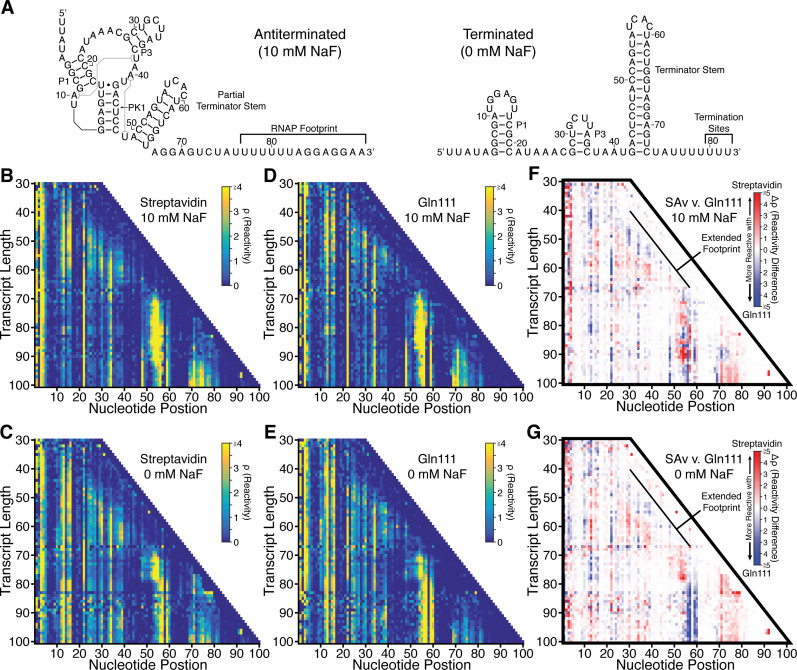
Cotranscriptional SHAPE-Seq of the *B. cereus crcB* fluoride riboswitch using biotin–SAv and Gln111 roadblocking. (**A**) Antiterminated and terminated secondary structures of the *B. cereus crcB* fluoride riboswitch. Structures are drawn according to phylogenetic (22), crystallographic (32) and cotranscriptional SHAPE-Seq (10) data. (**B** and **C**) Cotranscriptional SHAPE-Seq reactivity matrices produced using biotin–SAv roadblocking using 1xBiotin templates with 10 mM (B) or 0 mM (C) sodium fluoride (NaF). (**D** and **E**) Cotranscriptional SHAPE-Seq reactivity matrices produced using Gln111 roadblocking with 10 mM (D) or 0 mM (E) NaF (10). Gln111 data was downloaded from the RMDB (http://rmdb.stanford.edu/repository/) (42) (Supplementary Table S6). (**F** and **G**) Reactivity differences (Δρ) between biotin–SAv and Gln111 roadblocking data with 10 mM NaF (F) and 0 mM NaF (G). Regions of low reactivity tend to have Δρ values that are close to 0, whereas regions with moderate or high reactivity exhibit greater Δρ values. Results shown in (B) and (C) are *n* = 1 and are representative of four biological replicates (Supplementary Figure S1).

We next compared the cotranscriptional SHAPE-Seq reactivity measurements made for the *crcB* fluoride riboswitch using biotin–SAv roadblocks to previous measurements made using Gln111 roadblocks (10) (Supplementary Table S6). Our previous characterization of the *crcB* fluoride riboswitch revealed key signatures of aptamer folding and fluoride binding as well as the fluoride-dependent bifurcation of the RNA folding pathway to produce the riboswitch ‘ON’ and ‘OFF’ regulatory states (10) (Figure 5A). The same molecular signatures and their associated transitions are readily observable in reactivity matrices produced using biotin–SAv roadblocking (Figure 5B–E and Supplementary Figures S1A–B, S2A–B and S3A–B) indicating that overall, cotranscriptional SHAPE-Seq uncovers the same RNA structural information regardless of whether a biotin–SAv or Gln111 transcription roadblock is used.

We then compared biotin–SAv and Gln111 cotranscriptional SHAPE-Seq reactivity profiles for all RNA intermediates by calculating reactivity differences (Δ*ρ*) (Figure 5F–G and Supplementary Figures S2C–D and S3C–D). In perfect agreement with the analysis of precisely-positioned roadblocks (Figure 3D), Δ*ρ* analysis reveals that when biotin–SAv roadblocking is used, the RNAP footprint can protect an additional upstream ∼4–7 nt from SHAPE modification, as can be seen by the presence of a stripe of lower reactivity adjacent to the RNAP position compared to when Gln111 roadblocks are used. (Figure 5F–G and Supplementary Figures S2C–D and S3C–D). This behavior is remarkably consistent, and is visible in all biotin–SAv cotranscriptional SHAPE-Seq experiments, both in the presence and absence of fluoride.

**Figure 6.**
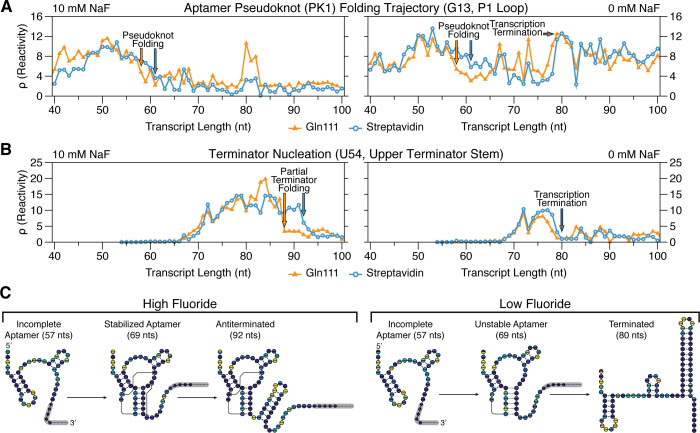
Comparison of *B. cereus crcB* fluoride riboswitch RNA folding transitions observed with biotin–SAv and Gln111 roadblocking. Gln111 data was downloaded from RMDB (http://rmdb.stanford.edu/repository/) (42) (Supplementary Table S6). (**A**) Cotranscriptional SHAPE-Seq reactivity trace of nucleotide G13 showing the fluoride-independent folding of pseudoknot PK1 followed by the fluoride-dependent bifurcation of the riboswitch folding pathway. (**B**) Cotranscriptional SHAPE-Seq reactivity trace of nucleotide U54 showing nucleation of the upper terminator stem. (**C**) *B. cereus crcB* fluoride riboswitch folding pathway. A model for the cotranscriptional folding pathway of the *B. cereus crcB* fluoride riboswitch in the presence (left) and absence (right) of NaF is shown. The transcript lengths shown are representative of key RNA structural states in the fluoride riboswitch folding pathway. Circles represent nucleotides and are colored according to cotranscriptional SHAPE-Seq reactivity obtained using SAv (top half of each circle) or Gln111 (bottom half of each circle) transcription roadblocks (Figure 5B–E). Full reactivity profiles for these lengths are shown in Supplementary Figure S5. Results shown are *n* = 1 and are representative of four biological replicates (Supplementary Figure S1).

Consistent with the interpretation that the collision of RNAP with a biotin–SAv roadblock produces backtracked complexes in different sequence contexts, RNA folding transitions associated with aptamer folding and terminator nucleation are displaced downstream by 1–4 transcript lengths and appear to be more gradual when TECs are stalled with biotin–SAv (Figure 6 and Supplementary Figures S1C, S2E–F and S3E–F). The first such major structural transition that is observed earlier is the fluoride-independent decrease in P1 loop (nt 11–16) reactivity as it pairs with the lower 6 nt of the terminator stem (nt 42–47) to form the pseudoknot PK1 (Figure 5A) (10). With Gln111, PK1 folds abruptly as P1 loop reactivity decreases sharply over transcript lengths 57–59, independent of fluoride concentration. With biotin–SAv, P1 loop reactivity decreases more gradually over transcript lengths 57–63, with an even more gradual drop in the absence of fluoride (Figure 6A and Supplementary Figures S1C, S2E and S3E). Furthermore, reactivity changes at nucleotides A10 and A22 that were previously shown to be associated with aptamer folding (10) are also displaced downstream in the biotin–SAv dataset such that they remain coordinated with PK1 folding (Supplementary Figure S4).

Following aptamer folding, the *crcB* fluoride riboswitch directs transcription termination or antitermination in the absence or presence of fluoride, respectively. In the absence of fluoride, terminator nucleation is observed as a coordinated reactivity decrease in the upper terminator stem (nt 52–55) and reactivity increase in the P1 loop as the terminator hairpin winds and disrupts PK1 (10). Terminator folding (35) in the absence of fluoride is consistently displaced downstream by 1 transcript length when biotin–SAv roadblocking is used, occurring across lengths 76–79 with Gln111 and 77–80 with streptavidin (Figure 6B and Supplementary Figures S1C, S2F and S3F). In the presence of fluoride, partial folding of the upper terminator stem is delayed until RNAP has traversed the poly-U tract and the fluoride-bound aptamer sequesters the base of the terminator stem so that only a partial terminator hairpin can form (10). In contrast to termination in the absence of fluoride, partial terminator nucleation in the presence of fluoride is more sensitive to the roadblock used, occurring at length 88 with Gln111 and length 92 with biotin–SAv (Figure 6B and Supplementary Figures S1C, S2F and S3F). The relative insensitivity of terminator hairpin folding to roadblock type when nucleation occurs in close proximity to RNAP is consistent with observations that nascent RNA structure can prevent RNAP backtracking (36). Importantly, all RNA structural states associated with the *crcB* fluoride riboswitch termination and antitermination transitions are identified by cotranscriptional SHAPE-Seq regardless of the transcription roadblock used (Supplementary Figure S5).

The final noteworthy distinction between cotranscriptional SHAPE-Seq reactivity profiles produced with biotin–SAv and Gln111 roadblocking is observed at the transcription termination sites in the presence of fluoride. Because *crcB* fluoride riboswitch antitermination efficiency in cotranscriptional SHAPE-Seq conditions is close to 100% (10), we do not expect to see reactivity signatures of transcription termination in the presence of fluoride. Nonetheless, previously we observed high P1 loop reactivity at the termination sites (80–82) when Gln111 is used to stall TECs (Figure 6A and Supplementary Figures S2E and S3E). In contrast, P1 loop reactivity remains low at the termination sites when biotin–SAv roadblocking is used, suggesting that TECs stalled by SAv are resistant to this effect.

Our cotranscriptional SHAPE-Seq analysis of the *crcB* fluoride riboswitch with biotin–SAv and Gln111 roadblocking demonstrates that the choice of transcription roadblock can influence the transcript length at which a transition is observed by 1–4 nts. This observation is consistent with measurements of RNAP location relative to the RNA 3΄ end made using precisely placed roadblocks (Figure 3), arguing that these measurements are generalizable to diverse sequence contexts. Thus, despite subtle distinctions in reactivity measurements, it is abundantly clear that both roadblocking strategies capture the same reactivity signatures associated with aptamer folding and the riboswitch regulatory decision (Supplementary Figures S5 and S6).

## DISCUSSION

We have developed a sequence-independent method for distributing of stalled TECs across a DNA template using biotin–SAv roadblocking and characterized the basic properties of biotin–SAv as an internal transcription roadblock. We have also benchmarked the use of biotin–SAv roadblocks in cotranscriptional SHAPE-Seq against data that was previously generated using Gln111. We found that cotranscriptional SHAPE-Seq results were largely independent of the roadblock strategy used as long as the relative propensity for each transcription roadblock to induce backtracking was taken into account. Indeed, the overall cotranscriptional SHAPE-Seq reactivity landscape of the fluoride riboswitch folding pathway is independent of the stalled TEC distribution strategy (Figure 5B–E and Supplementary Figures S1A–B, S2A–B and S3A–B). We therefore suggest that while the use of Gln111 roadblocking for cotranscriptional SHAPE-Seq provides greater accuracy than biotin–SAv roadblocking in mapping folding events to specific transcript lengths, the simplicity and reduced cost of biotin–SAv roadblocking makes it better suited for generation of full cotranscriptional SHAPE-Seq reactivity profiles.

The use of biotin–SAv to halt TECs at all positions across a DNA template provides several advantages over Gln111 in the context of cotranscriptional SHAPE-Seq that both simplify and reduce the cost of generating high-resolution profiles of RNA folding pathways. First, amplification of DNA template libraries for biotin–SAv roadblocking only requires two primers, while Gln111 roadblocking requires a large primer set whose costs exceeds that of the biotin-11-dNTPs required for randomly biotinylated DNA template preparation, especially for long RNA targets. Second, whereas Gln111 is not commercially available, the use of SAv as a transcription roadblock requires only readily available reagents, thereby improving the accessibility of the method.

While biotin–SAv roadblocking provides several experimental advantages for cotranscriptional SHAPE-Seq, in some cases the use of Gln111 may prove advantageous because of its properties as a transcription roadblock. For example, while distributed biotin–SAv roadblocks provide a simple and effective method for elucidating full RNA folding pathways, Gln111 roadblocking is better suited for the precise mapping of RNA structural rearrangements over small subsets of intermediate transcripts. Furthermore, a useful property of Gln111 roadblocking in the context of cotranscriptional SHAPE-Seq is that it facilitates the identification and removal of reads from roadblock run-through products. Because the incorporation of an EcoRI site into a DNA template introduces non-native sequence, any TECs that ‘run-through’ the roadblock will synthesize an RNA containing aberrant sequence and can therefore be discarded during read alignment. In contrast, every DNA template used for biotin–SAv roadblocking contains the entirety of the target RNA sequence and therefore, reads generated by TECs that run-through the first roadblock they encounter and stop at a subsequent roadblock are indistinguishable from those that were successfully halted by the first roadblock encountered. Because non-template strand biotin–SAv complexes halt transcription at low efficiency and template strand biotin–SAv complexes halt transcription with high, but imperfect, efficiency, it is important to consider how roadblock run-through might influence a particular experimental outcome. This distinction does not influence cotranscriptional SHAPE-Seq results in the context of the *B. cereus crcB* fluoride riboswitch, but is an important consideration when studying RNAs that may be sensitive to transcription pausing (37,38) because readthrough of a roadblock could function as a non-native pause.

There are multiple strategies that could be applied to circumvent or minimize this limitation of biotin–SAv roadblocking. The simplest approach would be to limit DNA template biotinylation such that the population of DNA templates containing more than one biotin modification is virtually nonexistent so that roadblock run-through almost always results in run-off transcription. However, because this strategy will yield a high proportion of non-biotinylated DNA templates it would be important to enrich for stalled complexes by purifying away run-off transcription products (Figure 2B) in order to maximize the number of reads from RNAs within roadblocked TECs. Alternatively, enrichment for high-efficiency template-strand roadblocks should be achievable by adapting a previously published strategy for the formation of heteroduplex DNA templates (39). The specific published strategy (39) is incompatible with the preparation of randomly biotinylated DNA templates because the desired DNA heteroduplex is purified by biotin–SAv mobility shift. However the general approach can be adapted to the formation of heteroduplexes in which only the template strand is biotinylated through the use of an alternative affinity tag, such as digoxigenin. The primary disadvantage to this strategy is that the essential protocol adaptations require additional costly reagents and an increased template preparation scale. In experiments where roadblock run-through is a major concern, the most effective solution is likely to use Gln111 or an alternative sequence-specific transcription roadblock (20,21) to characterize specific intermediate transcript lengths of interest after an initial characterization of the entire folding pathway using biotin–SAv.

The development of a second strategy for distributing halted TECs across a DNA template afforded us the opportunity to examine the influence of transcription roadblock properties on cotranscriptional SHAPE-Seq data. The broad agreement of *B. cereus crcB* fluoride riboswitch reactivity matrices generated with each method is indicative of a high degree of experimental reproducibility, even across roadblocking strategies (Figure 5 and Supplementary Figure S6). Specifically, our analysis of the *B. cereus crcB* fluoride riboswitch revealed key molecular signatures depicting aptamer folding, fluoride binding, and the fluoride-dependent bifurcation of the riboswitch folding pathway into the terminated and antiterminated functional modes regardless of the roadblock used. The primary distinction between cotranscriptional SHAPE-Seq measurements made using biotin–SAv and Gln111 roadblocking is that observed structural transitions are shifted upstream by 1–4 nts when SAv is used to roadblock TECs. This distinction corresponds to a degree of uncertainty in the location of RNAP relative to the 3΄ end of the nascent transcript and is a direct consequence of using the RNA 3΄ end to indicate RNAP location. While the RNA 3΄ end generally provides a close approximation of the location of RNAP on a DNA template, it is not a direct measurement of RNAP position due inherent variability of RNA 3΄ end position relative to RNAP (40). Because RNA must emerge from RNAP (41) before it can fold, changes in the location of RNAP relative to the RNA 3΄ end by processes such as backtracking would sequester RNA proximal to the RNAP exit channel while simultaneously displacing the RNA 3΄ end from the active center, resulting in an apparent downstream shift in structural transitions. Given the inherent flexibility of biotin–SAv roadblocks due to the necessity of tethering biotin to the DNA duplex by a linker, it is not surprising that RNAP position would be less defined following collision with biotin–SAv than following collision with the relatively static Gln111 roadblock. Indeed, precise measurement of RNAP footprint clearly indicates that biotin–SAv-stalled TECs are more prone to backtracking than Gln111-stalled TECs. The use of biotin–dNTPs with a shorter linker could reduce the flexibility of the roadblock, however, to our knowledge only biotin-11-dNTPs have complete commercial availability.

The distribution of TECs across all positions of a DNA template presents a technical challenge for which numerous solutions with unique advantages and disadvantages exist. Protein-based roadblocking strategies (9,18,20,21) are advantageous because they do not rely on the efficient incorporation of a modified nucleotide during transcription. Instead, protein roadblocks leverage the ubiquitous challenge of traversing a physical barrier along a DNA template and should therefore be generalizable to RNA polymerases beyond *E. coli* RNAP. The availability of biotin–SAv and Gln111 as transcription roadblocking strategies in cotranscriptional SHAPE-Seq allows users to tailor the method to specific experimental needs and expands the utility of a powerful RNA structure probing method.

## DATA AVAILABILITY

Raw sequencing data that support the findings of this study have been deposited in the Small Read Archive (SRA) (http://www.ncbi.nlm.nih.gov/sra) with the BioProject accession code PRJNA374354. Individual BioSample accession codes are available in Supplementary Table S3. SHAPE-Seq Reactivity Spectra generated in this work have been deposited in the RNA Mapping Database (RMDB) (http://rmdb.stanford.edu/repository/) (42). Accession codes and sample details are available in Supplementary Table S4. Source data for Figures 4 6 and Supplementary Figures S1–S6 are available with the paper online. Full versions of cropped gel images in Figures 2A, 3B and C are available with the paper online. All other data that support the findings of this paper are available from the corresponding author upon reasonable request.

## Supplementary Material

Supplementary Data

## References

[1] Cech T.R., Steitz J.A. The noncoding RNA revolution-trashing old rules to forge new ones. Cell. 2014; 157:77–94. 24679528 10.1016/j.cell.2014.03.008

[2] Strobel E.J., Watters K.E., Loughrey D., Lucks J.B. RNA systems biology: uniting functional discoveries and structural tools to understand global roles of RNAs. Curr. Opin. Biotechnol. 2016; 39:182–191. 27132125 10.1016/j.copbio.2016.03.019 PMC5098397

[3] Al-Hashimi H.M., Walter N.G. RNA dynamics: it is about time. Curr. Opin. Struct. Biol. 2008; 18:321–329. 18547802 10.1016/j.sbi.2008.04.004 PMC2580758

[4] Mustoe A.M., Brooks C.L., Al-Hashimi H.M. Hierarchy of RNA functional dynamics. Annu. Rev. Biochem. 2014; 83:441–466. 24606137 10.1146/annurev-biochem-060713-035524 PMC4048628

[5] Pan T., Sosnick T. RNA folding during transcription. Annu. Rev. Biophys. Biomol. Struct. 2006; 35:161–175. 16689632 10.1146/annurev.biophys.35.040405.102053

[6] Wong T.N., Pan T. RNA folding during transcription: protocols and studies. Methods Enzymol. 2009; 468:167–193. 20946770 10.1016/S0076-6879(09)68009-5

[7] Wong T.N., Sosnick T.R., Pan T. Folding of noncoding RNAs during transcription facilitated by pausing-induced nonnative structures. Proc. Natl. Acad. Sci. U.S.A. 2007; 104:17995–18000. 17986617 10.1073/pnas.0705038104 PMC2084285

[8] Perdrizet G.A. 2nd, Artsimovitch I., Furman R., Sosnick T.R., Pan T. Transcriptional pausing coordinates folding of the aptamer domain and the expression platform of a riboswitch. Proc. Natl. Acad. Sci. U.S.A. 2012; 109:3323–3328. 22331895 10.1073/pnas.1113086109 PMC3295289

[9] Frieda K.L., Block S.M. Direct observation of cotranscriptional folding in an adenine riboswitch. Science. 2012; 338:397–400. 23087247 10.1126/science.1225722 PMC3496414

[10] Watters K.E., Strobel E.J., Yu A.M, Lis J.T., Lucks J.B. Cotranscriptional folding of a riboswitch at nucleotide resolution. Nat. Struct. Mol. Biol. 2016; 23:1124–1131. 27798597 10.1038/nsmb.3316 PMC5497173

[11] Watters K.E., Yu A.M, Strobel E.J., Settle A.H., Lucks J.B. Characterizing RNA structures in vitro and in vivo with selective 2΄-hydroxyl acylation analyzed by primer extension sequencing (SHAPE-Seq). Methods. 2016; 103:34–48. 27064082 10.1016/j.ymeth.2016.04.002 PMC4921265

[12] Loughrey D., Watters K.E., Settle A.H., Lucks J.B. SHAPE-Seq 2.0: systematic optimization and extension of high-throughput chemical probing of RNA secondary structure with next generation sequencing. Nucleic Acids Res. 2014; 42:e165. 10.1093/nar/gku909 PMC4245970 25303992

[13] Lucks J.B., Mortimer S.A., Trapnell C., Luo S., Aviran S., Schroth G.P., Pachter L., Doudna J.A., Arkin A.P. Multiplexed RNA structure characterization with selective 2΄-hydroxyl acylation analyzed by primer extension sequencing (SHAPE-Seq). Proc. Natl. Acad. Sci. U.S.A. 2011; 108:11063–11068. 21642531 10.1073/pnas.1106501108 PMC3131332

[14] Weeks K.M., Mauger D.M. Exploring RNA structural codes with SHAPE chemistry. Acc. Chem. Res. 2011; 44:1280–1291. 21615079 10.1021/ar200051h PMC3177967

[15] Aviran S., Lucks J.B., Pachter L. Proc. 49th Allerton Conf. on Communication, Control, and Computing. 2011; Monticello 1743–1750.

[16] Bindewald E., Wendeler M., Legiewicz M., Bona M.K., Wang Y., Pritt M.J., Le Grice S.F., Shapiro B.A. Correlating SHAPE signatures with three-dimensional RNA structures. RNA. 2011; 17:1688–1696. 21752927 10.1261/rna.2640111 PMC3162334

[17] Wright D.J., King K., Modrich P. The negative charge of Glu-111 is required to activate the cleavage center of EcoRI endonuclease. J. Biol. Chem. 1989; 264:11816–11821. 2745418

[18] Pavco P.A., Steege D.A. Elongation by Escherichia coli RNA polymerase is blocked in vitro by a site-specific DNA binding protein. J. Biol. Chem. 1990; 265:9960–9969. 1693618

[19] Mortimer S.A., Weeks K.M. Time-resolved RNA SHAPE chemistry: quantitative RNA structure analysis in one-second snapshots and at single-nucleotide resolution. Nat. Protoc. 2009; 4:1413–1421. 19745823 10.1038/nprot.2009.126 PMC4950915

[20] Qi L.S., Larson M.H., Gilbert L.A., Doudna J.A., Weissman J.S., Arkin A.P., Lim W.A. Repurposing CRISPR as an RNA-guided platform for sequence-specific control of gene expression. Cell. 2013; 152:1173–1183. 23452860 10.1016/j.cell.2013.02.022 PMC3664290

[21] Tome J.M., Ozer A., Pagano J.M., Gheba D., Schroth G.P., Lis J.T. Comprehensive analysis of RNA-protein interactions by high-throughput sequencing-RNA affinity profiling. Nat. Methods. 2014; 11:683–688. 24809628 10.1038/nmeth.2970 PMC4073888

[22] Baker J.L., Sudarsan N., Weinberg Z., Roth A., Stockbridge R.B., Breaker R.R. Widespread genetic switches and toxicity resistance proteins for fluoride. Science. 2012; 335:233–235. 22194412 10.1126/science.1215063 PMC4140402

[23] Kaplan D.L., Davey M.J., O’Donnell M. Mcm4,6,7 uses a ‘pump in ring’ mechanism to unwind DNA by steric exclusion and actively translocate along a duplex. J. Biol. Chem. 2003; 278:49171–49182. 13679365 10.1074/jbc.M308074200

[24] Shin J.H., Jiang Y., Grabowski B., Hurwitz J., Kelman Z. Substrate requirements for duplex DNA translocation by the eukaryal and archaeal minichromosome maintenance helicases. J. Biol. Chem. 2003; 278:49053–49062. 12975364 10.1074/jbc.M308599200

[25] Buenrostro J.D., Araya C.L., Chircus L.M., Layton C.J., Chang H.Y., Snyder M.P., Greenleaf W.J. Quantitative analysis of RNA-protein interactions on a massively parallel array reveals biophysical and evolutionary landscapes. Nat. Biotechnol. 2014; 32:562–568. 24727714 10.1038/nbt.2880 PMC4414031

[26] Chauvier A., Picard-Jean F., Berger-Dancause J.C., Bastet L., Naghdi M.R., Dube A., Turcotte P., Perreault J., Lafontaine D.A. Transcriptional pausing at the translation start site operates as a critical checkpoint for riboswitch regulation. Nat. Commun. 2017; 8:13892. 28071751 10.1038/ncomms13892 PMC5234074

[27] Bae B., Feklistov A., Lass-Napiorkowska A., Landick R., Darst S.A. Structure of a bacterial RNA polymerase holoenzyme open promoter complex. Elife. 2015; 4, doi:10.7554/eLife.08504. 10.7554/eLife.08504 PMC4593229 26349032

[28] Nudler E. RNA polymerase backtracking in gene regulation and genome instability. Cell. 2012; 149:1438–1445. 22726433 10.1016/j.cell.2012.06.003 PMC3815583

[29] Borukhov S., Sagitov V., Goldfarb A. Transcript cleavage factors from E. coli. Cell. 1993; 72:459–466. 8431948 10.1016/0092-8674(93)90121-6

[30] Nedialkov Y.A., Burton Z.F. Translocation and fidelity of Escherichia coli RNA polymerase. Transcription. 2013; 4:136–143. 23863783 10.4161/trns.25527 PMC4042587

[31] Tasara T., Angerer B., Damond M., Winter H., Dorhofer S., Hubscher U., Amacker M. Incorporation of reporter molecule-labeled nucleotides by DNA polymerases. II. High-density labeling of natural DNA. Nucleic Acids Res. 2003; 31:2636–2646. 12736314 10.1093/nar/gkg371 PMC156052

[32] Ren A., Rajashankar K.R., Patel D.J. Fluoride ion encapsulation by Mg2+ ions and phosphates in a fluoride riboswitch. Nature. 2012; 486:85–89. 22678284 10.1038/nature11152 PMC3744881

[33] Hafner M., Renwick N., Brown M., Mihailovic A., Holoch D., Lin C., Pena J.T., Nusbaum J.D., Morozov P., Ludwig J. RNA-ligase-dependent biases in miRNA representation in deep-sequenced small RNA cDNA libraries. RNA. 2011; 17:1697–1712. 21775473 10.1261/rna.2799511 PMC3162335

[34] Alon S., Vigneault F., Eminaga S., Christodoulou D.C., Seidman J.G., Church G.M., Eisenberg E. Barcoding bias in high-throughput multiplex sequencing of miRNA. Genome Res. 2011; 21:1506–1511. 21750102 10.1101/gr.121715.111 PMC3166835

[35] Ray-Soni A., Bellecourt M.J., Landick R. Mechanisms of bacterial transcription termination: all good things must end. Annu. Rev. Biochem. 2016; 85:319–347. 27023849 10.1146/annurev-biochem-060815-014844

[36] Artsimovitch I., Landick R. Interaction of a nascent RNA structure with RNA polymerase is required for hairpin-dependent transcriptional pausing but not for transcript release. Genes Dev. 1998; 12:3110–3122. 9765211 10.1101/gad.12.19.3110 PMC317188

[37] Hein P.P., Kolb K.E., Windgassen T., Bellecourt M.J., Darst S.A., Mooney R.A., Landick R. RNA polymerase pausing and nascent-RNA structure formation are linked through clamp-domain movement. Nat. Struct. Mol. Biol. 2014; 21:794–802. 25108353 10.1038/nsmb.2867 PMC4156911

[38] Zhang J., Landick R. A two-way street: regulatory interplay between RNA polymerase and Nascent RNA structure. Trends Biochem. Sci. 2016; 41:293–310. 26822487 10.1016/j.tibs.2015.12.009 PMC4911296

[39] Ring B.Z., Yarnell W.S., Roberts J.W. Function of E. coli RNA polymerase sigma factor sigma 70 in promoter-proximal pausing. Cell. 1996; 86:485–493. 8756730 10.1016/s0092-8674(00)80121-x

[40] Artsimovitch I., Landick R. Pausing by bacterial RNA polymerase is mediated by mechanistically distinct classes of signals. Proc. Natl. Acad. Sci. U.S.A. 2000; 97:7090–7095. 10860976 10.1073/pnas.97.13.7090 PMC16504

[41] Komissarova N., Kashlev M. Functional topography of nascent RNA in elongation intermediates of RNA polymerase. Proc. Natl. Acad. Sci. U.S.A. 1998; 95:14699–14704. 9843952 10.1073/pnas.95.25.14699 PMC24512

[42] Cordero P., Lucks J.B., Das R. An RNA Mapping DataBase for curating RNA structure mapping experiments. Bioinformatics. 2012; 28:3006–3008. 22976082 10.1093/bioinformatics/bts554 PMC3496344

