# Supplementary material for: Distributed biotin–streptavidin transcription roadblocks for mapping cotranscriptional RNA folding
Source: Nucleic Acids Res. 2017 Apr 8;45(12):e109. doi: 10.1093/nar/gkx233 (PMC5499547; doi:10.1093/nar/gkx233)
Supplement: Supplementary Data [file gkx233_supp.zip › nar-00129-met-g-2017-File008.pdf]

Supplementary Materials for

Distributed Biotin-Streptavidin Transcription Roadblocks for Mapping Cotranscriptional RNA Folding

Eric J. Strobel, Kyle E. Watters, Yuri Nedialkov, Irina Artsimovitch, and Julius B. Lucks

This pdf file includes:

Supplementary Figures S1 to S6
Supplementary Tables S1 to S6
Supplementary Note S1

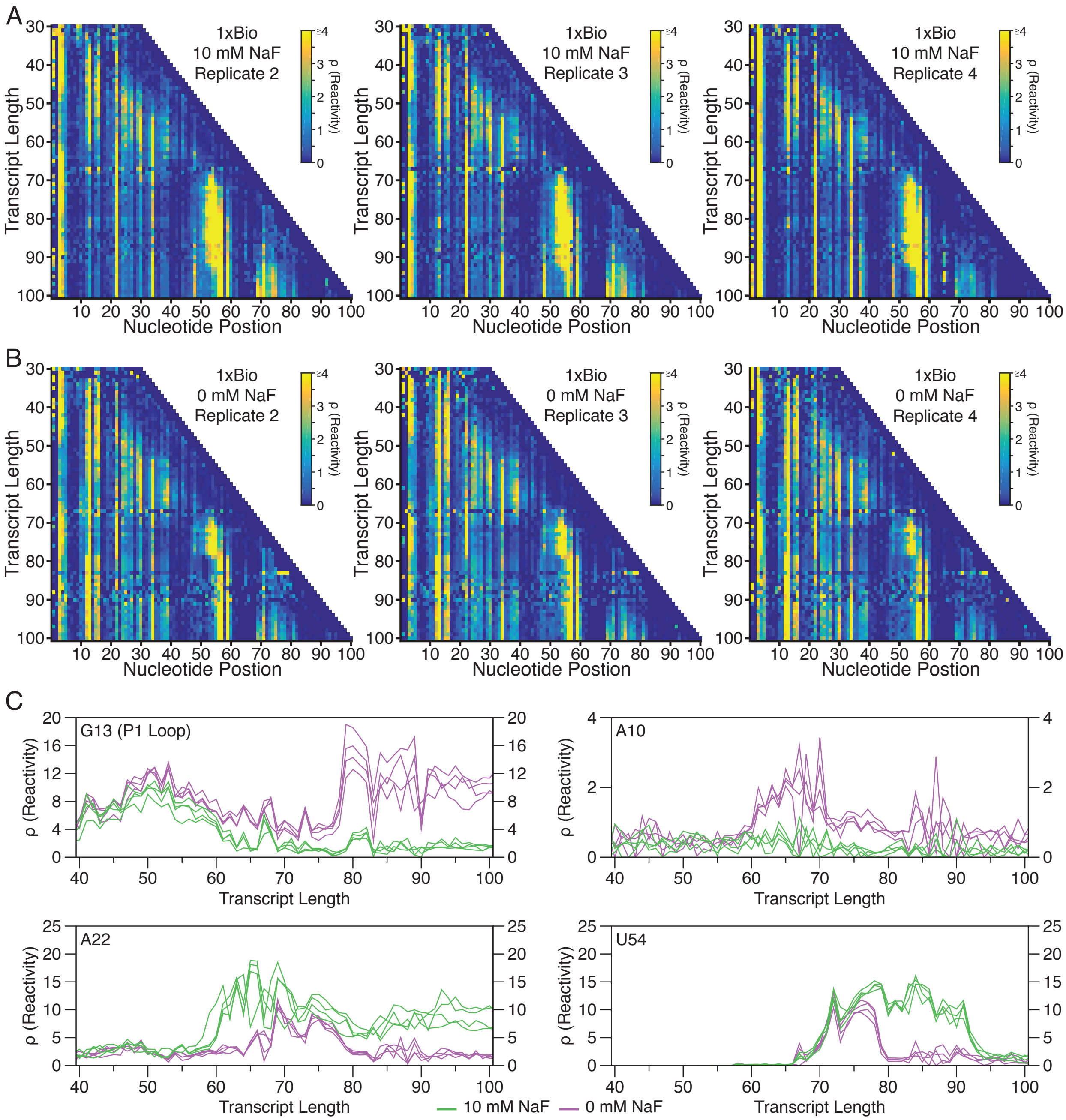

Supplementary Figure S1. Additional replicates of cotranscriptional SHAPE-Seq Data for the *B*. *cereus crcB* fluoride riboswitch using 1x biotinylated DNA template. Additional three replicates for the *crcB* fluoride riboswitch cotranscriptional SHAPE-Seq Data with **(A)** 10 mM NaF and **(B)** 0 mM NaF. **(C)** Replicate reactivity traces for nt G13, A10, A22, and U54 in the presence and absence of fluoride. Data from Figures 5B-C is also

included alongside the replicates in (A) and (B) and Supplementary FIgures S1A, and S1B.

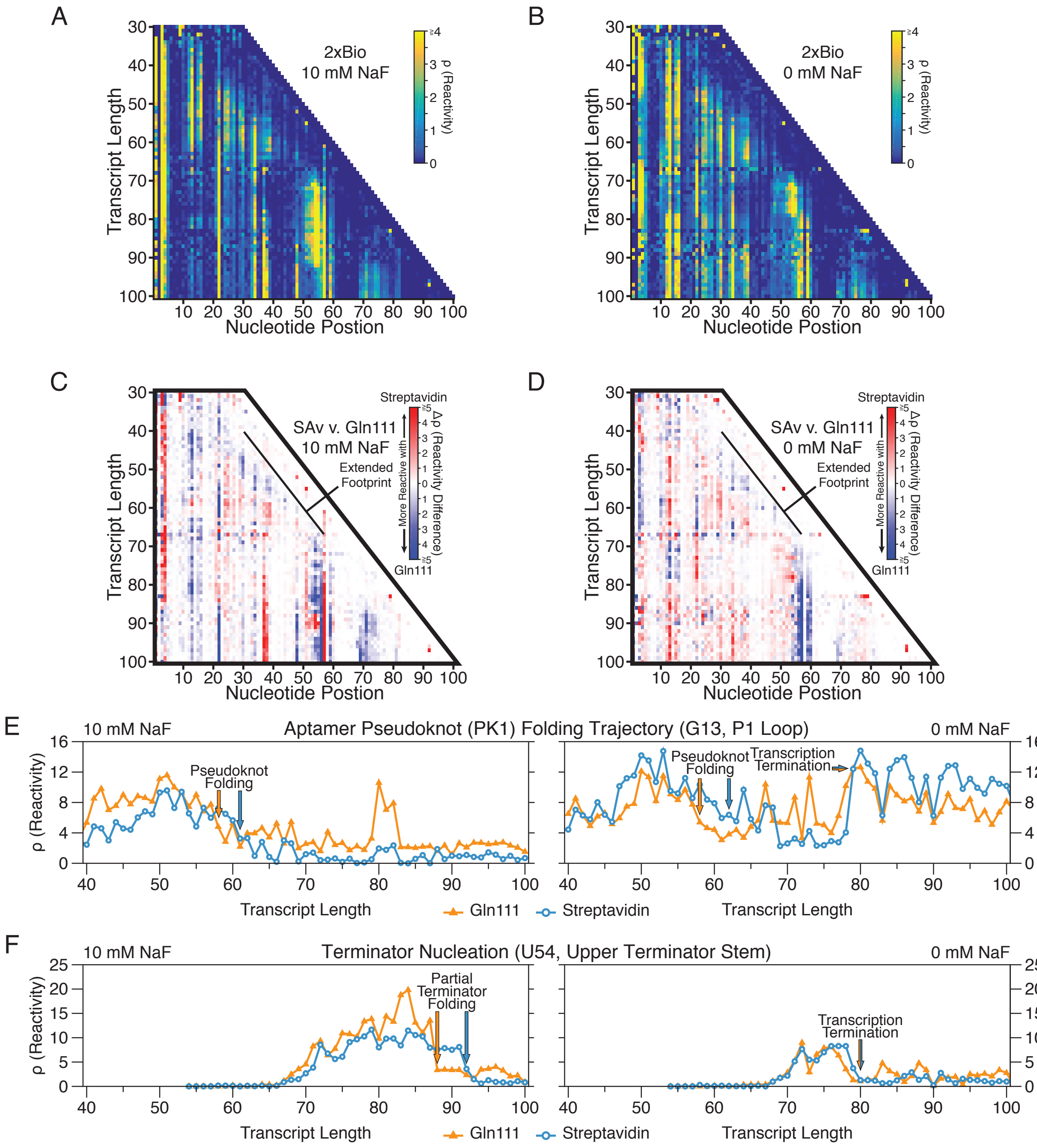

Supplementary Figure S2. Comparing cotranscriptional SHAPE-Seq reactivity profiles of the *B. cereus crcB* fluoride riboswitch using streptavidin (2x biotinylated template) with Gln111 roadblocking. Gln111 data was downloaded from the RNA Mapping Database (RMDB) (http://rmdb.stanford.edu/repository/) (Cordero et al. 2012) (Supplementary Table S6). **(A-B)** Cotranscriptional SHAPE-Seq reactivity matrices

produced using 2x streptavidin roadblocking with 10 mM (A) and 0 mM (B) NaF. **(C-D)** Reactivity differences (Δρ) between streptavidin and Gln111 roadblocking data with 10 mM (C) and 0 mM NaF(D). **(E)** Reactivity trace of nucleotide G13 showing the fluoride-independent folding of pseudoknot PK1 and the fluoride-dependent bifurcation of the riboswitch folding pathway. Key transitions are annotated. **(F)** Reactivity trace of nucleotide U54 showing nucleation of the upper terminator stem. Key transitions are annotated. Results shown are n=1.

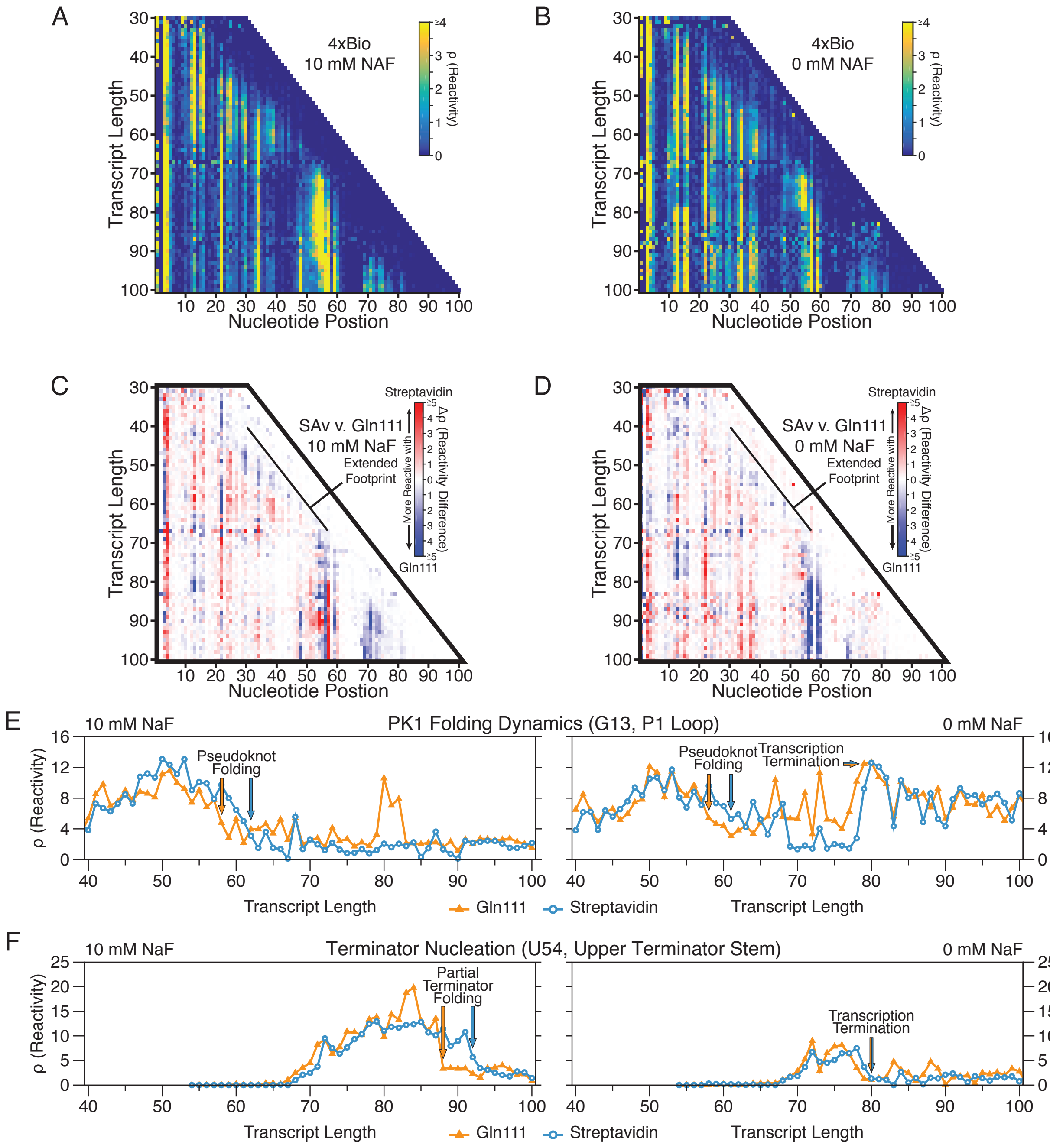

Supplementary Figure S3. Comparing cotranscriptional SHAPE-Seq reactivity profiles of the *B. cereus crcB* fluoride riboswitch using streptavidin (4x biotinylated template) with Gln111 roadblocking. Gln111 data was downloaded from RMDB (http://rmdb.stanford.edu/repository/) (Cordero et al. 2012) (Supplementary Table S6). Data presented as in Supplementary Figure S2. Results shown are n=1.

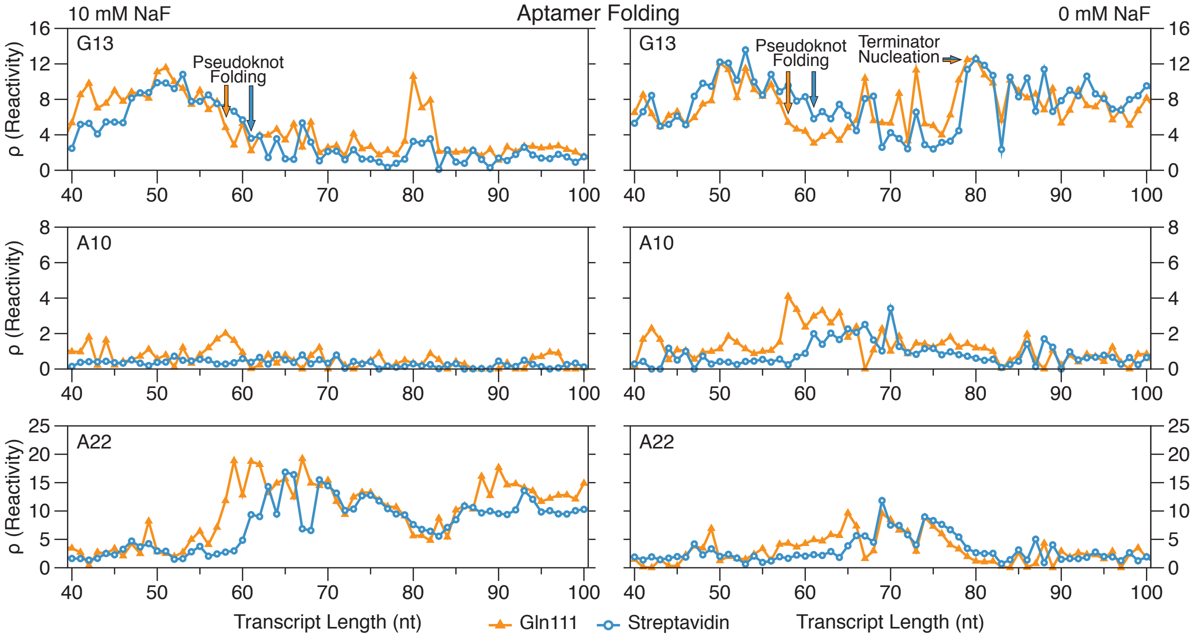

Supplementary Figure S4. Coordinated displacement of aptamer folding reactivity transitions associated with streptavidin roadblocking. Streptavidin data (1x biotin) is from Figure 5B-C. Gln111 data was downloaded from the RMDB (http://rmdb.stanford.edu/repository/) (Cordero et al. 2012) (Supplementary Table S6). Reactivity traces of nucleotides G13, A10, and A22 show the fluoride-independent folding of the *crcB* pseudoknot (G13), fluoride-dependent stabilization of the aptamer (A10, A22) and the fluoride-dependent bifurcation of the riboswitch folding pathway (G13). Reactivity changes at nucleotides A10 and A22 that are associated with pseudoknot formation and stabilization remain coordinated with aptamer folding regardless of the transcript length at which pseudoknot folding is observed. Data from Figures. 5B-C are n=1 and are representative of four biological replicates (Supplementary Figure S1).

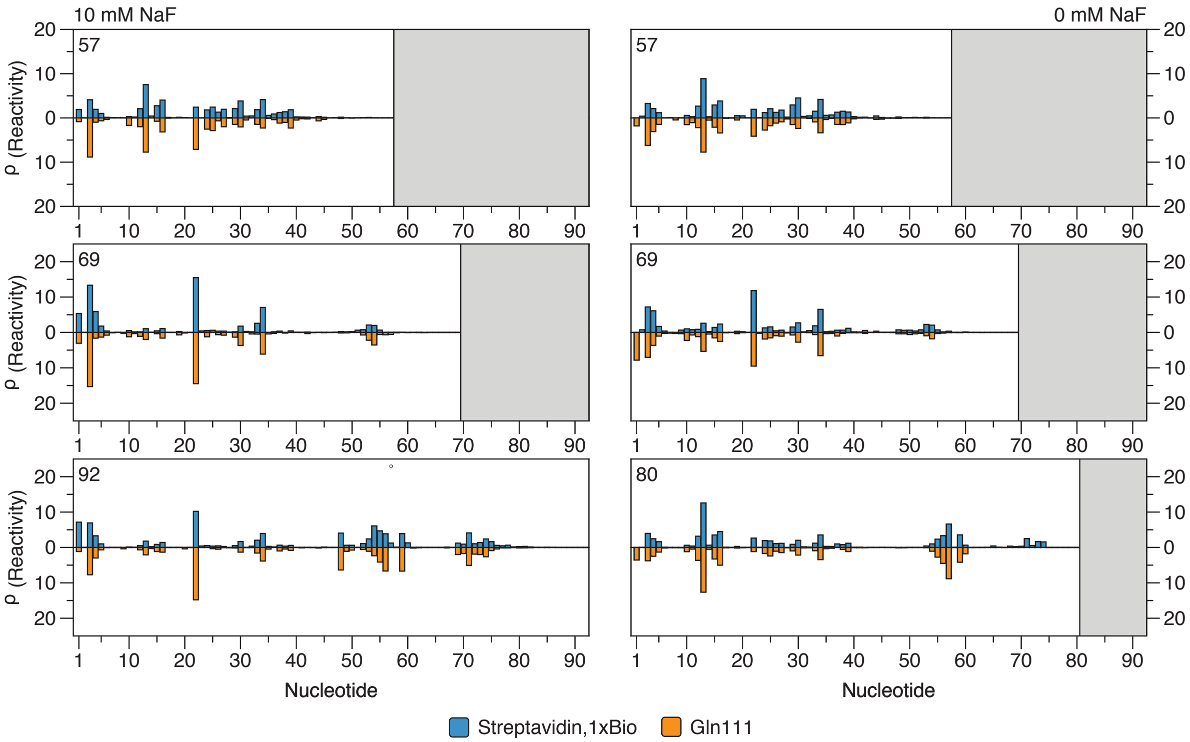

Supplementary Figure S5. Cotranscriptional SHAPE-Seq identifies the RNA structural states associated with *crcB* aptamer folding with both streptavidin and Gln111 transcription roadblocks. Cotranscriptional SHAPE-Seq with 10 mM NaF reactivity profiles for transcript lengths 57, 69, and 92 (antiterminated) are shown. Cotranscriptional SHAPE-Seq with 0 mM NaF reactivity profiles for transcript lengths 57, 69, and 80 (terminated) are shown. Streptavidin data is taken from Figure 5B-C. Gln111 data was downloaded from RMDB (http://rmdb.stanford.edu/repository/) (Cordero et al. 2012) (Supplementary Table S6). Data from Figure 5B-C are n=1 and are representative of four biological replicates (Supplementary Figure S1).

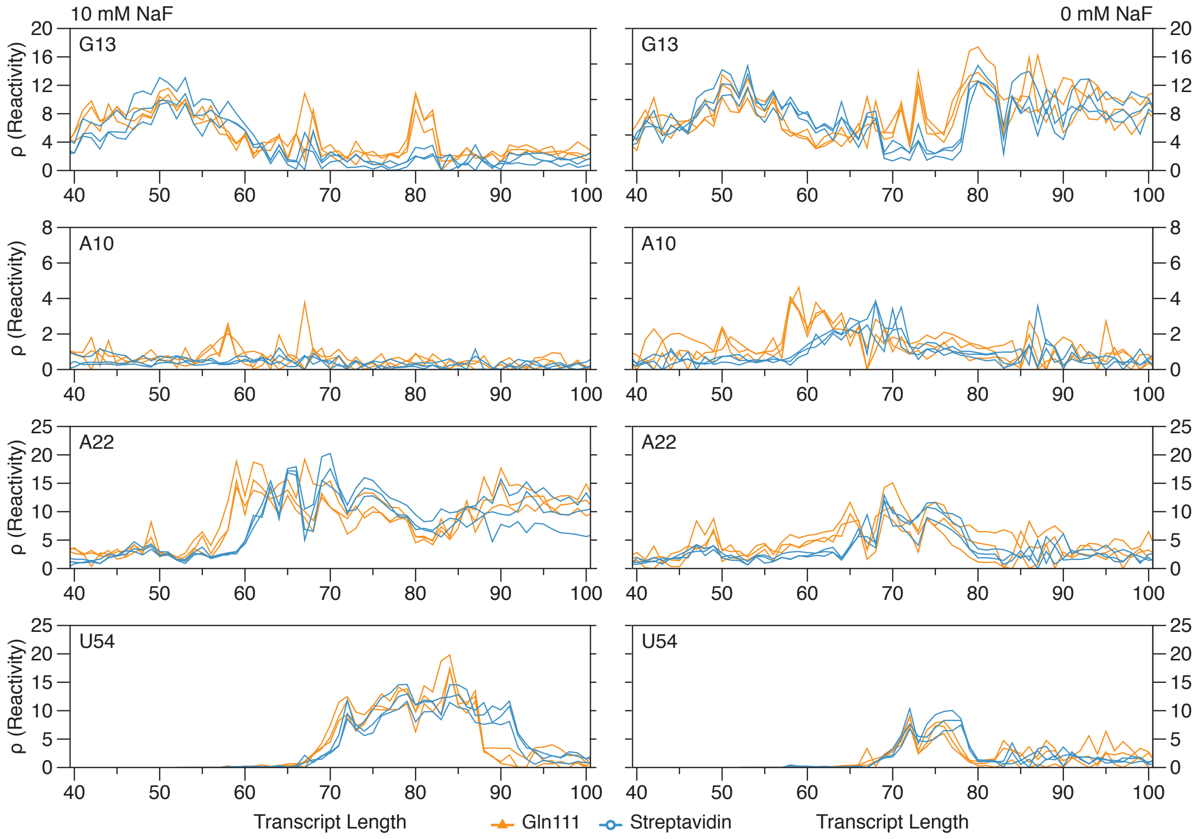

Supplementary Figure S6. Reproducibility of cotranscriptional SHAPE-Seq reactivity profiles with streptavidin and Gln111 transcription roadblocks. Streptavidin data is from Figs. 5B, 5C, S2A, S2B, S3A, and S3B. Gln111 data was downloaded from RMDB (http://rmdb.stanford.edu/repository/) (Cordero et al. 2012) (Supplementary Table S6). Reactivity traces of nucleotide G13, A10, A22, and U54 are shown. Results shown are n=1. Data from Figs. 5B and 5C are representative of four biological replicates (Supplementary Figure S1).

Supplementary Table S1. Oligonucleotides used for DNA template amplification or assembly.

Below is a table of oligonucleotides used for the preparation of *in vitro* transcription DNA templates. Abbreviations within primer sequences are as follows: '/iBiodT/' is a biotin modified thymidine residue with a linker length of 11, '/5Biosg/' is a 5' biotin moiety. These abbreviations were used for compatibility with the Integrated DNA Technologies ordering notation.

**Description**	**Sequence**	**ID**
Forward primer for template strand biotin modification DNA templates	aaatgtagcacctgaagtcagcccc	A
Reverse primer for SRP DNA template w/ template strand position 33 biotin dT modification	TGACCTGGTAAACAGAG/iBiodT/AGCGTTGCGGGAGAACC	B
Reverse primer for SRP DNA template w/ template strand position 42 biotin dT modification	TTCCGGACCTGACCTGG/iBiodT/AAACAGAGTAGCGTTGC	C
Nontranscribed strand for SRP DNA template w/ template strand position 33 biotin modification	CTAAAGATCTTTGACAGCTAGCTCAGTCCTAGGTATAATGAATTCATCGGGGGCTCTGTTGGTTCTCCCGCAACGCT/iBiodT/CTCTGTTTACCA	D
Transcribed strand for SRP DNA template w/ template strand position 33 biotin modification	TGGTAAACAGAGAAGCGTTGCGGGAGAACCAACAGAGCCCCCGATGAATTCATTATACCTAGGACTGAGCTAGCTGTCAAAGATCTTTAG	E
Forward primer for amplification of randomly biotinylated DNA templates with promoter J23119	CTAAAGATCTTTGACAGCTAGCTCAGTCCTAGGTATAATACTAGT	F
Reverse primer for amplification of DNA templates with terminal biotin; primes from SF-GFP	/5Biosg/CAACAAGAATTGGGACAACTCCAGTG	G
Forward primer for amplification of pIA226; λP_R promoter	CGTTAAATCTATCACCGCAAGG	H
Reverse primer for amplification of pIA226; introduces EcoRI site	ATGTCTTCCAGCACACATCGGAATTCAGACTAGTCAGG	I
Reverse primer for amplification of pIA226; template strand biotin modification	CTTCCAGCACACATCGCCTGGAATTC/iBiodT/AGTCAGGATG	J
Reverse primer for amplification of DNA templates without terminal biotin; primes from SF-GFP	CAACAAGAATTGGGACAACTCCAGTG	K

Supplementary Table S2. DNA templates used for in vitro transcription.

Sequences of DNA templates used for *in vitro* transcription. Signal Recognition Particle (SRP) RNA sequence is as described in Wong *et al.* (6). A ribosome binding site (RBS) and superfolder GFP (SFGFP) sequence was included downstream of the *B. cereus crcB* fluoride riboswitch. Promoter sequences are blue. The position of internal biotin dT modifications are in red. EcoRI site is in green.

Description	Sequence
SRP w/ internal biotin-dT in template strand at +33	AAATGTAGCACCTGAAGTCAGCCCCATACGATATAAGTTGTAATTCTCATGTTTGACAGCT<br>TATCATCGATAAGCTTCCGATGGCGCGCCGAGAGGCTTTACACTTTATGCTTCCGGCTGAA<br>TTCTAAAGATCTTTGACAGCTAGCTCAGTCCTAGGTATAATGAATTCATCGGGGGCTCTGT<br>TGGTTCTCCCGCAACGCTACTCTGTTTACCAGGTCA
SRP w/ internal biotin-dT in template strand at +42	AAATGTAGCACCTGAAGTCAGCCCCATACGATATAAGTTGTAATTCTCATGTTTGACAGCT<br>TATCATCGATAAGCTTCCGATGGCGCGCCGAGAGGCTTTACACTTTATGCTTCCGGCTGAA<br>TTCTAAAGATCTTTGACAGCTAGCTCAGTCCTAGGTATAATGAATTCATCGGGGGCTCTGT<br>TGGTTCTCCCGCAACGCTACTCTGTTTACCAGGTCAGGTCCGGAA
SRP w/ internal biotin-dT in nontemplate strand at +33	CTAAAGATCTTTGACAGCTAGCTCAGTCCTAGGTATAATGAATTCATCGGGGGCTCTGTTG<br>GTTCTCCCGCAACGCTTCTCTGTTTACCA
Bacillus cereus crcB Fluoride Riboswitch Template	CTAAAGATCTTTGACAGCTAGCTCAGTCCTAGGTATAATACTAGTTTATAGGCGATGGAGT<br>TCGCCATAAACGCTGCTTAGCTAATGACTCCTACCAGTATCACTACTGGTAGGAGTCTATT<br>TTTTTAGGAGGAAGGATCTATGAGCAAAGGAGAAGAACTTTTCACTGGAGTTGTCCCAATT<br>CTTGTTG
pIA226 w/ EcoRI Recognition Site	CGTTAAATCTATCACCGCAAGGGATAAATATCTAACACCGTGCGTGTTGACTATTTTACCT<br>CTGGCGGTGATAATGGTTGCATGTAGTAAGGAGGTTGTATGGAAGACGTTCCTCATCATCA<br>CCATCATCCTGACTAGTCTGAATTCCGATGTGTGCTGGAAGACAT
pIA226 w/ internal biotin-dT in template strand at +56	CGTTAAATCTATCACCGCAAGGGATAAATATCTAACACCGTGCGTGTTGACTATTTTACCT<br>CTGGCGGTGATAATGGTTGCATGTAGTAAGGAGGTTGTATGGAAGACGTTCCTCATCATCA<br>CCATCATCCTGACTAGAATTCCAGGCGATGTGTGCTGGAAG

Supplementary Table S3. Small Read Archive (SRA) deposition table.

All primary sequencing data generated in this work are freely available from the Small Read Archive (http://www.ncbi.nlm.nih.gov/sra), accessible via the BioProject accession number PRJNA374354, or using the individual accession numbers below.

SRA Accession	RNA	Experiment	Figure(s)
SAMN06456610	F- riboswitch, wt, 1xBiotin Template (replicate 1)	0 mM NaF cotranscriptional	Figs. 4, 5, 6, S1, S4, S5, S6
SAMN06456611	F- riboswitch, wt, 2xBiotin Template (replicate 1)	0 mM NaF cotranscriptional	Figs. 4, S2, S6
SAMN06456612	F- riboswitch, wt, 4xBiotin Template (replicate 1)	0 mM NaF cotranscriptional	Figs. 4, S3, S6
SAMN06456613	F- riboswitch, wt, 1xBiotin Template (replicate 1)	10 mM NaF cotranscriptional	Figs. 4, 5, 6, S1, S4, S5, S6
SAMN06456614	F- riboswitch, wt, 2xBiotin Template (replicate 1)	10 mM NaF cotranscriptional	Figs. 4, S2, S6
SAMN06456615	F- riboswitch, wt, 4xBiotin Template (replicate 1)	10 mM NaF cotranscriptional	Figs. 4, S3, S6
SAMN06456616	F- riboswitch, wt, 1xBiotin Template (replicate 2)	0 mM NaF cotranscriptional	Fig. S1
SAMN06456617	F- riboswitch, wt, 1xBiotin Template (replicate 3)	0 mM NaF cotranscriptional	Fig. S1
SAMN06456618	F- riboswitch, wt, 1xBiotin Template (replicate 4)	0 mM NaF cotranscriptional	Fig. S1
SAMN06456619	F- riboswitch, wt, 1xBiotin Template (replicate 2)	10 mM NaF cotranscriptional	Fig. S1
SAMN06456620	F- riboswitch, wt, 1xBiotin Template (replicate 3)	10 mM NaF cotranscriptional	Fig. S1
SAMN06456621	F- riboswitch, wt, 1xBiotin Template (replicate 4)	10 mM NaF cotranscriptional	Fig. S1

Supplementary Table S4. RMDB data deposition table.

SHAPE-Seq reactivity spectra generated in this work are freely available from RMDB (http://rmdb.stanford.edu/repository/) (Cordero et al. 2012), accessible using the RMDB ID numbers indicated in the table below.

RMDB ID	RNA	Experiment	Figure(s)
FLUOR_BZCN_0021	F- riboswitch, wt, 1xBiotin Template (replicate 1)	0 mM NaF cotranscriptional	Figs. 5, 6, S1, S4, S5, S6
FLUOR_BZCN_0022	F- riboswitch, wt, 2xBiotin Template (replicate 1)	0 mM NaF cotranscriptional	Figs. S2, S6
FLUOR_BZCN_0023	F- riboswitch, wt, 4xBiotin Template (replicate 1)	0 mM NaF cotranscriptional	Figs. S3, S6
FLUOR_BZCN_0024	F- riboswitch, wt, 1xBiotin Template (replicate 1)	10 mM NaF cotranscriptional	Figs. 5, 6, S1, S4, S5, S6
FLUOR_BZCN_0025	F- riboswitch, wt, 2xBiotin Template (replicate 1)	10 mM NaF cotranscriptional	Figs. S2, S6
FLUOR_BZCN_0026	F- riboswitch, wt, 4xBiotin Template (replicate 1)	10 mM NaF cotranscriptional	Figs. S3, S6
FLUOR_BZCN_0027	F- riboswitch, wt, 1xBiotin Template (replicate 2)	0 mM NaF cotranscriptional	Fig. S1
FLUOR_BZCN_0028	F- riboswitch, wt, 1xBiotin Template (replicate 3)	0 mM NaF cotranscriptional	Fig. S1
FLUOR_BZCN_0029	F- riboswitch, wt, 1xBiotin Template (replicate 4)	0 mM NaF cotranscriptional	Fig. S1
FLUOR_BZCN_0030	F- riboswitch, wt, 1xBiotin Template (replicate 2)	10 mM NaF cotranscriptional	Fig. S1
FLUOR_BZCN_0031	F- riboswitch, wt, 1xBiotin Template (replicate 3)	10 mM NaF cotranscriptional	Fig. S1
FLUOR_BZCN_0032	F- riboswitch, wt, 1xBiotin Template (replicate 4)	10 mM NaF cotranscriptional	Fig. S1

Supplementary Table S5. Accession numbers for previously published sequencing data.

Sequencing data from Watters et al., 2016 used in this work were accessed through the Small Read Archive (http://www.ncbi.nlm.nih.gov/sra) BioProject accession number PRJNA342175. Individual BioSample Accession numbers are listed below:

SRA Accession	RNA	Experiment	Figure(s)
SRX2159297	F- riboswitch, wt (replicate 1)	no ligand, cotranscriptional	Fig. 4D
SRX2159317	F- riboswitch, wt (replicate 1)	10 mM ligand, cotranscriptional	Fig. 4D

Table S6. Accession numbers for previously published reactivity data.

Cotranscriptional SHAPE-Seq reactivity spectra from Watters et al., 2016 that were used in this work were accessed in the RNA Mapping Database (RMDB) (http://rmdb.stanford.edu/repository/) (Cordero et al. 2012), using the RMDB ID numbers indicated in the table below.

RMDB ID	RNA	Experiment	Figure(s)
FLUORSW_BZCN_0001	F- riboswitch, wt (replicate 1)	no ligand, cotranscriptional	Figs. 5, 6, S2, S3, S4 & S5, &S6
FLUORSW_BZCN_0002	F- riboswitch, wt (replicate 2)	no ligand, cotranscriptional	Figs. S6
FLUORSW_BZCN_0003	F- riboswitch, wt (replicate 3)	no ligand, cotranscriptional	Figs. S6
FLUORSW_BZCN_0004	F- riboswitch, wt (replicate 1)	10 mM ligand, cotranscriptional	Figs. 5, 6, S2, S3, S4, S5, & S6
FLUORSW_BZCN_0005	F- riboswitch, wt (replicate 2)	10 mM ligand, cotranscriptional	Figs. S6
FLUORSW_BZCN_0006	F- riboswitch, wt (replicate 3)	10 mM ligand, cotranscriptional	Figs. S6

Supplementary Note 1. Precise measurement of RNAP position using ExoIII mapping

A 'GAATTC' EcoRI recognition site and an internal biotin dT were precisely positioned such that RNAP stalls at +45 on each DNA template. Because the DNA template encoding an EcoRI site is 86 bp long, 41 bp are expected to be downstream of the RNAP active center after roadblock collision. Similarly, because the DNA template containing a biotin dT is 82 bp, 37 bp are expected to be downstream of the RNAP active site after roadblock collision. When the RNAP footprint is taken into account, it is anticipated that non-backtracked TECs that are roadblocked by Gln111 and biotin-SAv will create a barrier to ExoIII digestion 55 and 51 nt from the template DNA strand 5' end, respectively. When TECs are roadblocked by Gln111, we observe a barrier at 55 and 56 nt, indicating that RNAP does not backtrack more than 1 nt after collision with Gln111. In contrast, when TECs are roadblocked by biotin-SAv, we observe an 'expanded' RNAP footprint with a barrier at 54–56 nt, suggesting that RNAP backtracks by as many as 5 nt following collision with biotin-SAv.
