# Supplementary material for: Distributed biotin–streptavidin transcription roadblocks for mapping cotranscriptional RNA folding
Source: Nucleic Acids Res. 2017 Apr 8;45(12):e109. doi: 10.1093/nar/gkx233 (PMC5499547; doi:10.1093/nar/gkx233)

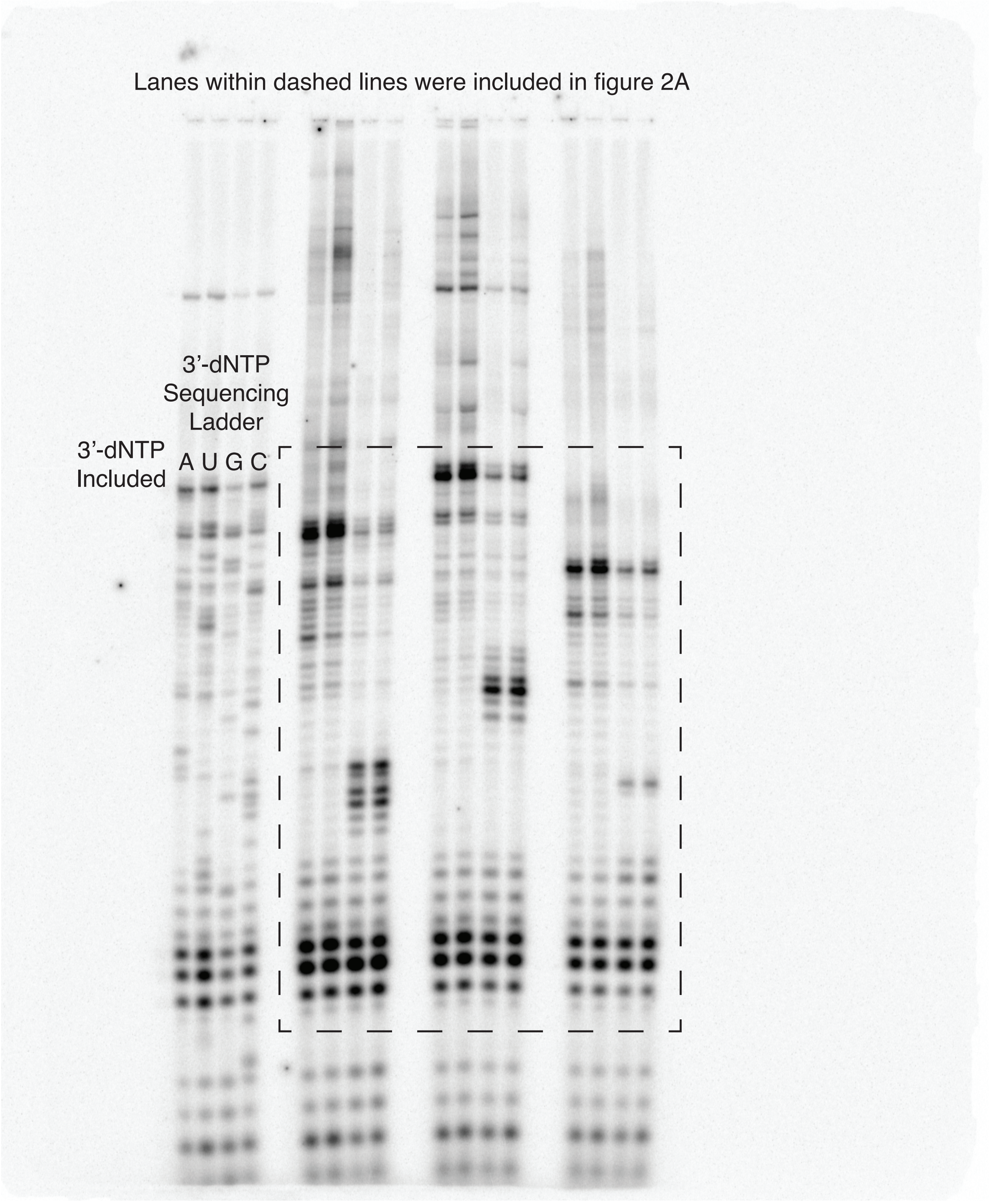
Lanes within dashed lines were included in figure 2A
3'-dNTP Sequencing Ladder
3'-dNTP Included
A U G C

Lanes within dashed lines were included in figure 3B

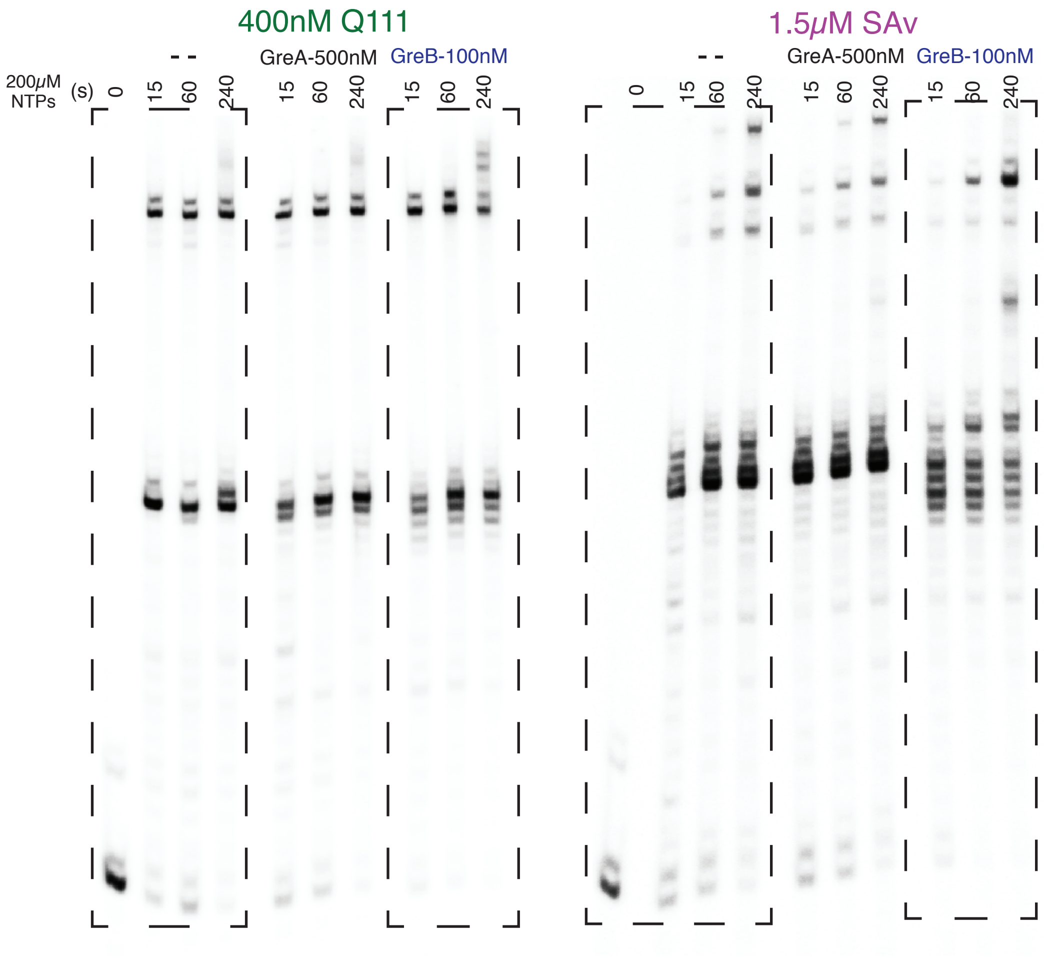

Lanes within dashed lines were included in figure 3C

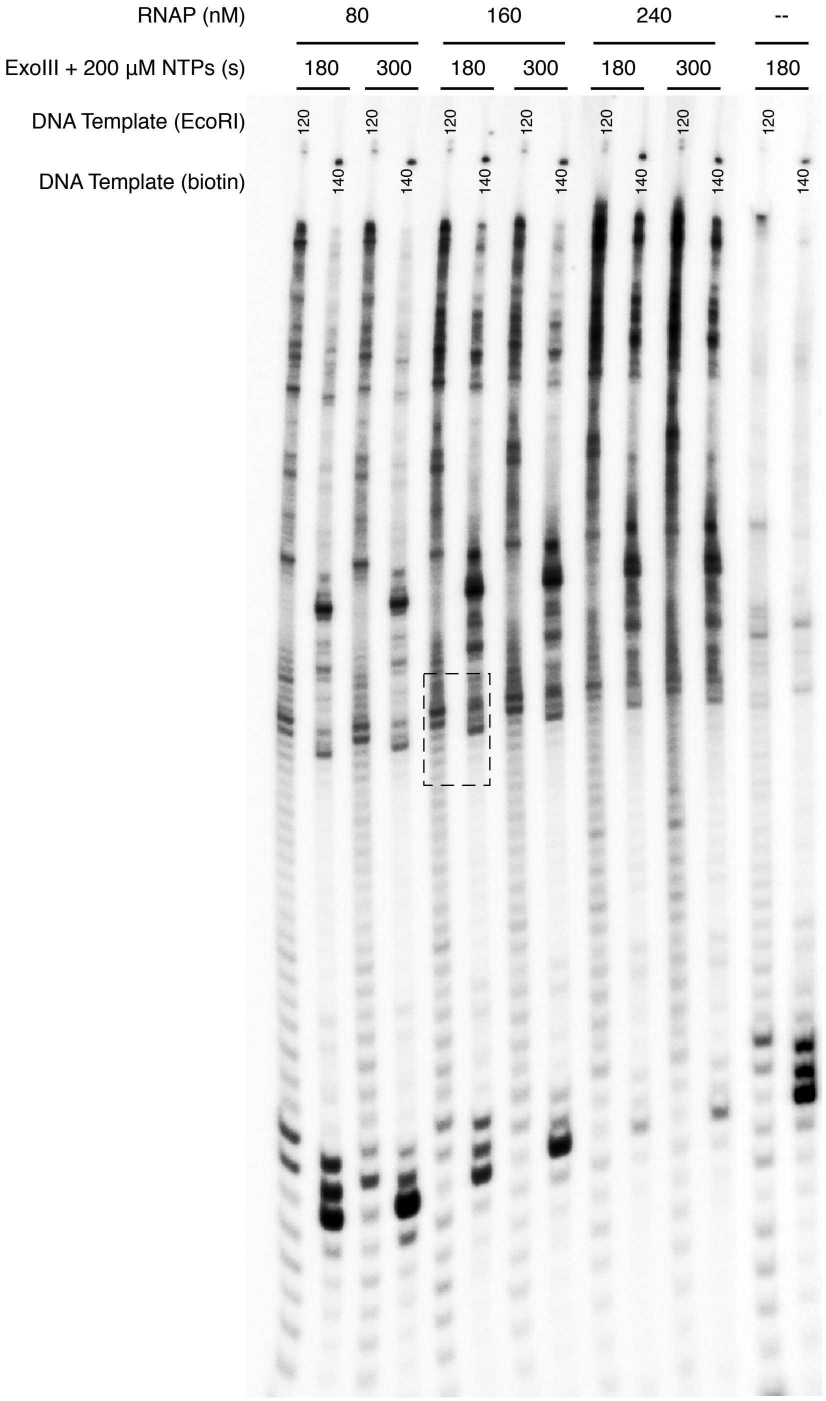

Supplement: Supplementary Data [file gkx233_supp.zip › nar-00129-met-g-2017-File018.pdf]
